# Symbiosis, dysbiosis and the impact of horizontal exchange on bacterial microbiomes in higher fungus-gardening ants

**DOI:** 10.1038/s41598-024-53218-6

**Published:** 2024-02-08

**Authors:** Blake Bringhurst, Matthew Greenwold, Katrin Kellner, Jon N. Seal

**Affiliations:** 1 Department of Biology, University of Texas at Tyler, 3900 University Blvd, Tyler, TX 757998 USA; 2 Department of Evolution, Ecology and Organismal Biology, The Ohio State University, 1315 Kinnear Rd, Columbus, OH 43212 USA

**Keywords:** Coevolution, Microbial communities, Fungi

## Abstract

Advances in our understanding of symbiotic stability have demonstrated that microorganisms are key to understanding the homeostasis of obligate symbioses. Fungus-gardening ants are excellent model systems for exploring how microorganisms may be involved in symbiotic homeostasis as the host and symbionts are macroscopic and can be easily experimentally manipulated. Their coevolutionary history has been well-studied; examinations of which have depicted broad clade-to-clade specificity between the ants and fungus. Few studies hitherto have addressed the roles of microbiomes in stabilizing these associations. Here, we quantified changes in microbiome structure as a result of experimentally induced horizontal exchange of symbionts. This was done by performing cross-fostering experiments forcing ants to grow novel fungi and comparing known temporally unstable (undergoing dysbiosis) and stable combinations. We found that fungus-gardening ants alter their unstable, novel garden microbiomes into configurations like those found in native gardens. Patterns of dysbiosis/symbiosis appear to be predictable in that two related species with similar specificity patterns also show similar patterns of microbial change, whereas a species with more relaxed specificity does not show such microbiome change or restructuring when growing different fungi. It appears that clade-to-clade specificity patterns are the outcomes of community-level interactions that promote stability or cause symbiotic collapse.

## Introduction

One of the key advances of the 21st century is the realization that symbioses are stable and persist across evolutionary time scales and are often significant to the generation and maintenance of biodiversity^1–6^. Another major discovery is that microorganisms are important to understanding the homeostasis of complex, multi-cellular eukaryotes^7–13^. Symbioses often consist of more than a single host and a single symbiont, and are best viewed as a community of interacting and perhaps coevolving macro- and microorganisms (the microbiome)^14–19^. One of the central issues facing the study of symbioses is understanding how these complex entities are organized and function across ecological and evolutionary scales and especially how microbial and macrobiological components interact.

Fungus–gardening ants (subtribe Attina) are an example of a symbiosis that is best viewed as a community of interacting partners. Not only does the interaction consist of ants and their fungus garden, but rather the main macroscopic partners are associated with hundreds if not thousands of bacteria and microfungi^20–25^. The most heavily studied microbial symbionts within the attine ant system are bacteria that are thought to have defensive functions, especially Actinobacteria (*Pseudonocardia*, *Streptomyces* and *Amycolatopsis*) and *Burkholderia*^26–29^. Such bacteria are suggested to aid in the suppression of parasitic fungi that consume the fungus garden by producing secondary metabolites that function as antibiotics^28,30^. Other bacterial associates of the symbiosis appear to have a nutritional function. For example, *Klebsiella* and *Pantoea* in leaf–cutter ant fungal gardens are known to fix atmospheric nitrogen with the nitrogen assimilated into ant biomass^31^. Additionally, members of Rhizobiales have been found in the gut lumen of *Acromyrmex* ants and are suspected to fix nitrogen through use of nitrogenase proteins^32^. Entomoplastamales, such as strains of *Mesoplasma* and *Spiroplasma*, are symbionts of leaf–cutter ants and have been suspected of converting arginine into NH_3_ within the ant, allowing the ant to “fertilize” their fungal gardens with their feces^33^. On the other hand, the roles of Entomoplastamales as mutualists of the fungus gardens are less clear. High abundances of *Mesoplasma* have been shown to be more likely to cause fungal garden failure in the leaf-cutting ant *Atta texana*, even though high abundances of *Mesoplasma* have been found in healthy, established fungus gardens of *Trachymyrmex septentrionalis* and *Mycetomoellerius turrifex*^34–37^. Although considerable information exists on the importance of these bacteria that appear involved in the movement of nutrients through the symbiosis, there is little understanding how these taxa may be influenced by ant and fungal macrosymbionts.

Within the most derived lineages of Attina (the so-called higher attines), there are two broad classes of ants and fungi. There are the leaf–cutting ants (*Atta, Acromyrmex,* and *Amoimyrmex*) and the non-leaf–cutting ants (*Trachymyrmex*, *Mycetomoellerius*, *Sericomyrmex* and *Paratrachymyrmex*), with the fungi grown by these two groups consisting of two clades: Clade-A and Clade-B^38,39^. Clade-A fungi consists of a single described ‘species’ *Leucoagaricus gongylophorus*, while Clade-B conservatively consists of five clades of undescribed *Leucoagaricus* species^40,41^. Though leaf–cutting ants typically grow Clade-A fungus and the non–leaf–cutting ants grow mainly Clade-B fungi, there are some exceptions, as some populations of *T. arizonensis* are known to grow both clades and other leaf-cutting species have been found growing Clade-B fungi^39,41,42^.

The mechanisms that maintain the specificity between these groups of ants and fungi (or permit its relaxation) are currently not understood, though potentially arise from several factors. Specificity has been proposed to be maintained by ants actively preferring certain fungi and mechanisms that may involve fungal manipulation of the ants and unknown reasons that appear physiological^43–47^. For example, if *T. septentrionalis*, a species that is not known to grow Clade-A fungi, does not actively reject a Clade-A symbiont, they will grow the Clade-A fungus at a similar rate to Clade-B fungus, until ultimately the colonies growing Clade-A fungus experience a sudden, unrecoverable dramatic collapse^47–49^. Although the nature of the sudden decline and death of novel gardens suggested disease or some physiological incompatibility, the exact mechanisms causing decline were unknown. Different physiologies of fungal lineage were not likely an explanation either as stable combinations of other species exhibited strong host signals, suggesting that ants are interacting with fungal biology in some unknown way that influences the outcome of the interaction^41,42,50^. One possibility was that unstable combinations were associated with a loss of a crucial ingredient (possibly a bacteria or secondary metabolite, among other possibilities) as after catastrophic losses of fungal symbionts, replacement fungi either were unsuccessfully adopted or were repeatedly lost after reintroduction^48^. In the following paper, we examined whether bacterial microbiomes were involved in symbiosis or dysbiosis (defined here as the disruption of symbiotic relationships) by comparing the bacterial communities of ants and fungi that were forced to grow either Clade-A or Clade-B fungi. Some of these combinations were considered ‘novel’ as some species are not known to grow both clades of fungi.

## Methods

### Study species

The three *Trachymyrmex* species (*T. arizonensis*, *T. septentrionalis* and *T. pomonae*) in this study are commonly found throughout oak-pine forests in southern North America. *Trachymyrmex arizonensis* is found within oak-pine woodlands or dry scrublands in mid elevations (1000-2000 m) of the Chihuahuan and Sonoran Deserts in Arizona, New Mexico, Texas and the Mexican states of Chihuahua and Sonora^51^. *Trachymyrmex pomonae* co-occurs with *T. arizonensis* in dry exposed ridges of mid elevation oak-pine woodlands^51,52^. *Trachymyrmex septentrionalis* occurs in oak-pine savannas along the southeastern coastal plain of North America, from central Texas to Long Island New York, and is found inland to at least Kansas and Illinois^51,53,54^. *T. pomonae* and *T. septentrionalis* typically associate with Clade-B fungi, especially with fungi in so-called Clade-B4^40^. On the other hand, at least within the population found within the Chiricahua Mountains, *T. arizonensis* is known to associate both with Clade-B4 and Clade-A (*Leucoagaricus gongylophorus*) fungi^41^ (Seal, J. N. unpublished data).

### Set–up of *Trachymyrmex arizonensis*

**Figure 1:**
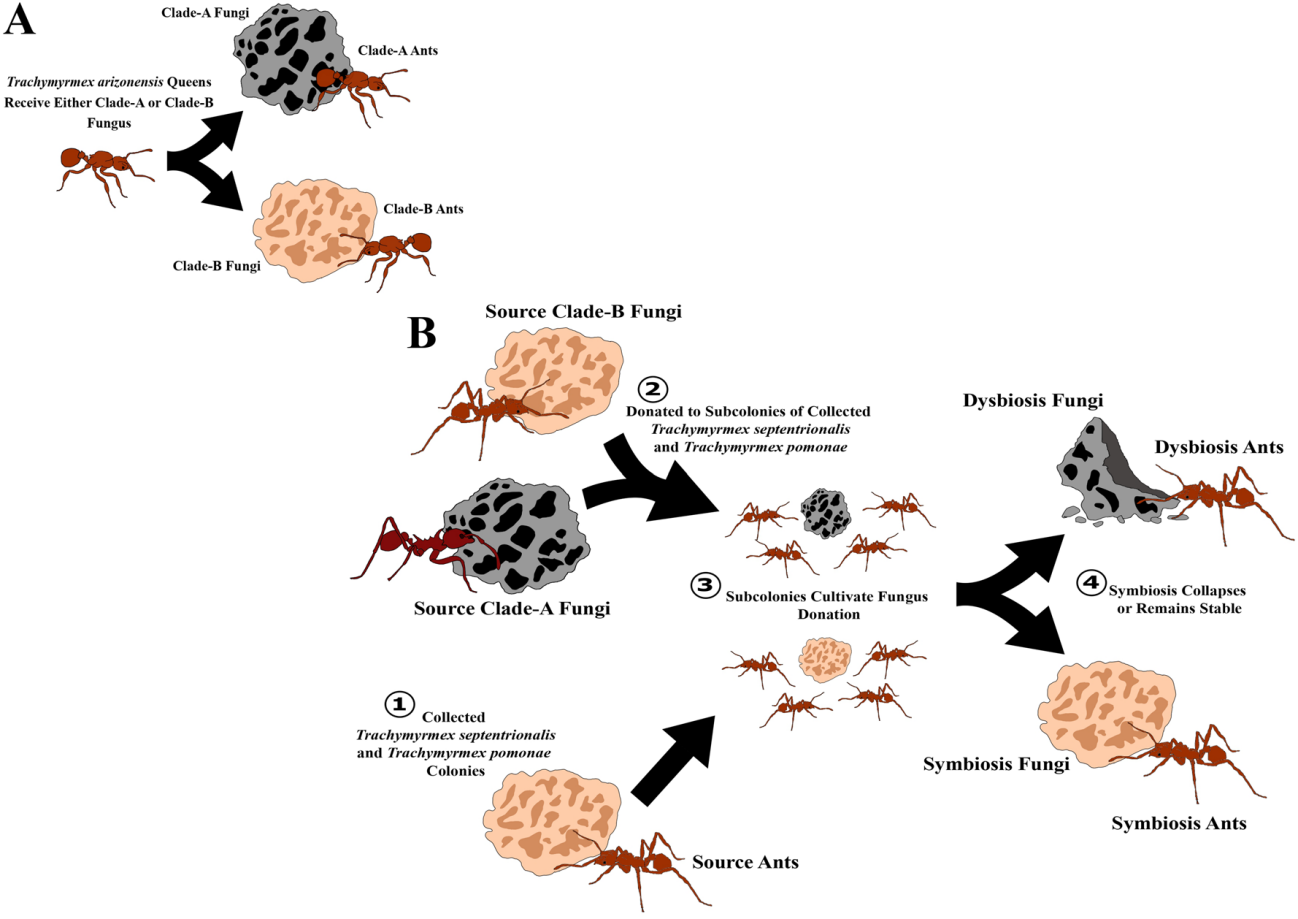
The differences in experimental design between (**A**) *Trachymyrmex arizonensis* queens and (**B**) *T. septentrionalis* and *T. pomonae* subcolonies. (**A**) *T. arizonensis* queens were collected and provided native Clade-A or Clade-B fungi. The *T. arizonensis* queens were then allowed to grow their colony utilizing their provided fungal cultivar, resulting in samples of ants taken from colonies growing stable Clade-A (Clade-A Ants) or Clade-B (Clade-B Ants) fungi. (**B**) Two subcolonies from colonies of *T. septentrionalis* and *T. pomonae* were created and provided either native, Clade-B fungi or novel, Clade-A fungi. The subcolonies were then allowed to grow their donated cultivars until the Clade-A gardens collapsed. Samples of the ants and fungal gardens were then taken to compare between symbiotic, Clade-B fungal and associated ant microbiomes and dysbiotic, Clade-A fungal and ant associated microbiomes. The figure was generated using Inkscape version 1.0 (https://inkscape.org/release/inkscape-1.0/).

The *T. arizonensis* colonies used in this study were reared from newly mated queens collected near Southwestern Research Station in southeastern Arizona (approximately 31°53' N, 109°13' W) in July 2011 and were previously utilized in Seal *et al*. (2014). Queens were either forced to grow a Clade-A fungus (n = 5) or Clade-B fungus (n = 6), with the species able to grow both as native cultivars (or a cultivar the species is known to grow naturally) (Supplementary Methods: Donor Fungus Sources) (Figure 1A). Forceps were ethanol–flame sterilized between donations. These colonies had been in the laboratory for at least five years before their microbiomes were sampled.

### Set–up of *Trachymyrmex pomonae* and *Trachymyrmex septentrionalis*

The *T. pomonae* colonies (n = 10) used in this study were collected during the summer of 2019 within the grounds of Southwestern Research Station. Colonies had since been maintained in the laboratory at the University of Texas at Tyler. Colonies of *T. septentrionalis* (n=12) were collected from the University of Texas at Austin Stengl “Lost Pines” Biological Research Station in Bastrop, Texas (approximately 30°05' N, 97°10' W) between April 8–9, 2021. These source colonies were allowed a week to adjust to lab conditions before further experimentation.

Two subcolonies were made from each collected source colony, with the queen (if collected) and fungus garden left with the original source colony (Figure 1B). Between 5 and 20 workers of *T. pomonae* and 10–20 workers of *T. septentrionali*s were removed from each source colony (with this number depending on the size of the original colony to ensure that it could still function without these workers) and placed in nesting dishes (Supplementary Table S1). The subcolonies were provided donations of either novel, Clade-A fungus (*T. pomonae* n = 10; *T. septentrionalis* n = 12) or native, Clade-B fungus (*T. pomonae* n = 10; *T. septentrionalis* n =12) (Supplementary Methods: Donor Fungus Sources). Fungal donations consisted of giving each subcolony a small snippet of fungus, about the size of a forcep tip. Forceps were ethanol–flame sterilized between donations. If any additional nuclei of fungus were observed within the nesting dishes away from the donated fungus, they were removed to ensure the growth of only the donated fungus and not any potential remnants of the fungus grown by the original colony stowed away on the ants. Clippings of the grown gardens were sequenced to confirm the ants were growing their provided cultivars (Supplementary Methods: Fungus Clade Confirmation).

The subcolonies were then allowed to grow their cultivars and were cared for every other day (Supplementary Methods: Subcolony Care). As the Clade-A fungus gardens of *T. pomonae* and *T. septentrionalis* were expected to undergo dysbiosis and collapse^48,55^, the health and productivity of each subcolony were monitored weekly. The dimensions (length, width, and height) of the fungal gardens were measured to determine their volume and any changes in the garden volumes over time. As the subcolonies varied in the number of initial workers provided, the average volume (mm^3^) of fungus per worker for the subcolonies given Clade-A and Clade-B cultivars were calculated for each measurement time point. Repeated measures ANOVAs were conducted in RStudio 4.2.2 to compare the growth between cultivars, with pairwise ANOVAs utilized as a post–hoc test to determine significant differences at certain time points. Permutational multivariate analysis of variance (PERMANOVA) tests were run using the vegan 2.6–4 R package to evaluate differences in microbiome structure between subcolonies growing the same fungus clade that collapsed and those that appeared stable by the end of the study^56^.

### Microbiome sampling

Samples for microbiome analysis of *T. pomonae* and *T. septentrionalis* ants and fungus were taken prior to donation and during the collapse (loss) of the fungus garden in colonies growing Clade-A fungi. Samples of Clade-B fungus and ants growing Clade-B fungus were taken at the same time points to allow for a valid comparison with their respective Clade-A associated samples. Further details on the sampling process for *T. septentrionalis* and *T. pomonae* can be found in Supplementary Methods: Microbiome Sampling Details. As the *T. arizonensis* colonies had been established in the laboratory for approximately ten years before these experiments and showed no sign of collapse, no source ants or fungus samples were taken^42^. Microbiome samples for *T. arizonensis* were taken at approximately five years after the queens had established their colonies in the laboratory. These samples consisted of a single whole ant and small snippet of fungus. All microbiome samples were collected using sterilized ethanol–flamed forceps, with the ant or fungus trimming placed in screw–capped vials filled with 400 μL of 100% ethanol. Samples were then stored in a –80 °C freezer until DNA extraction and sequencing.

### Microbiome analyses

DNA extraction and sequencing of the 16S samples was performed at MR DNA in Shallowater, Texas (http://www.mrdnalab.com/) and utilized the 27F 5’AGRGTTTGATCMTGGCTCAG and 519R 5’GTNTTACNGCGGCKGCTG primers, with these extraction methods and similar sequencing methods having been previously reported^34^. Microbiome sequences from MR DNA were processed using Qiime2–2020.6 (Qiime2) utilizing the same pipeline as Bringhurst *et al*., (2023), resulting in an OTU table for each species^57^. Sequences that were classified as chloroplasts, mitochondria, or unassigned were manually removed from the OTU tables (Supplementary Methods: Bacterial Distribution). OTU abundances for samples associated with each species were rarefied, using the GUniFrac 1.7 R package, at differing sampling depths to minimize the number of samples removed due to them not containing enough OTU abundances to meet the sampling depth threshold (*T. arizonensis* = 1000, *T. pomonae* = 380, *T. septentrionalis* = 700) (Supplementary Methods: Bacterial Distribution)^58^. The reduced sample sizes from samples not reaching these rarefaction thresholds (see Results) were utilized for statistical analyses.

### Quantification and statistical analyses

For alpha diversity comparisons, Shannon’s diversity index (H*'*) was calculated for each ant and fungus sample using the vegan 2.6-4 R package^56^. The mean Shannon’s diversity index values of the respective sample types were found and then compared amongst the ant or fungal sample types using Kruskal–Wallis rank sum test to indicate any significant differences (Supplementary Fig. S1). Dunn’s tests were performed, using the FSA 0.9.3 R package, as the post–hoc analysis to indicate which Shannon’s diversity index means were significantly different for comparisons with three or more means (Supplementary Fig. S1)^59^.

The cumulative percent abundances of each OTU for each sample type were utilized to make taxonomic bar plots for the source and post-donation samples for the ants and fungus. Taxonomic bar plots for each individual sample were also created to show the variation between samples (Supplementary Figs. S2–S6). In order to establish how the microbiomes of the source and post-donation samples are similar for the ants and fungus, Venn diagrams were constructed utilizing the eulerr 7.0.0 R package to highlight the number of shared taxa (Supplementary Figs. S7–S9)^60^. Non–metric multidimensional scaling (NMDS) plots based off of Bray Curtis distances were made using the vegan 2.6-4 R package to visually contrast the microbiomes of the source and post-donation samples for the ants and fungus^56^. PERMANOVA tests were run using the vegan 2.6–4 R package to compare the microbiomes of the source and post-donation samples for the ants and fungus to see if they were significantly different^56^. In order to compare *T. septentrionalis* and *T. pomonae* subcolonies derived from the same original colony, Bray Curtis dissimilarity index was calculated using the vegan 2.6-4 R package between the microbiomes of ants from the same source colony (comparing source, symbiotic, and dysbiotic ants) and between the microbiomes of gardens grown by ants from the same source colony (comparing symbiotic and dysbiotic fungi) (Supplementary Tables S2–S5)^56^.

In order to describe which taxa were causing the differences between the sample types determined by the PERMANOVA tests, two different analyses were used. Similarity percentages (SIMPER) analyses were run utilizing the vegan 2.6–4 R package to indicate which taxa contributed the most to the dissimilarity between sample types^56,61^. SIMPER analyses work in tandem with permutational significance tests (such as PERMANOVAs), with the permutational significance tests determining if the communities between sample types are significantly different and the SIMPER analysis determining which OTUs are driving the differences between sample types. Only the OTUs with the highest contribution to dissimilarity between comparisons and that add up to 70% of the cumulative dissimilarity were reported, as this appeared consistent with other papers on ant microbiomes^62,63^. Additionally, the indicspecies R package was used to conduct the indicator species analyses (ISA) to examine which taxa contribute most toward the overall variation in microbiome structure of the source and post-donation samples^64^.

## Results

### Subcolony growth and collapse

**Figure 2:**
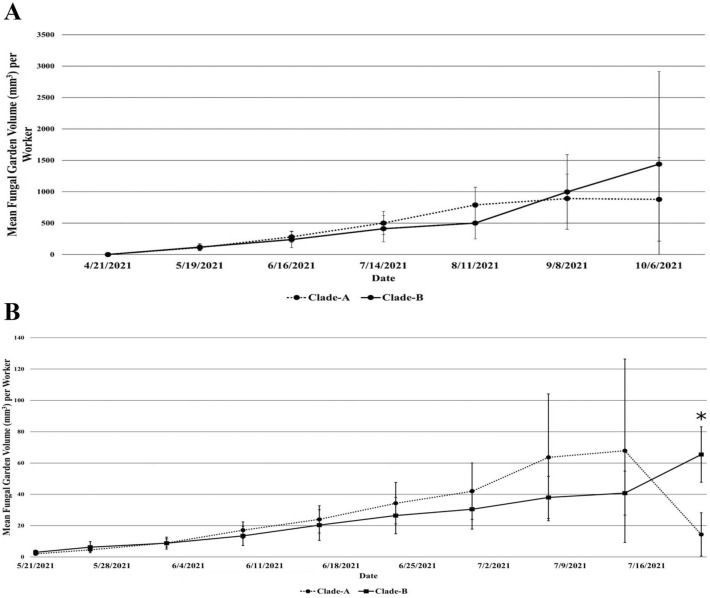
Mean fungal garden volumes (mm^3^) per ant over time for both Clade–A (dysbiotic) and Clade–B (symbiotic) (**A**) *Trachymyrmex septentrionalis* and (**B**) *T. pomonae* subcolonies. Error bars denote the 95% confidence interval around the mean. Symbols denote significant differences (*p* < 0.05) between the growths of Clade–A and Clade–B fungal gardens from the pairwise ANOVAs, where the p–values were adjusted using Bonferroni correction. (**A**) The volume of the fungal garden grew significantly over the course of the experiment (repeated measures ANOVA: F_1.44,15.82_= 8.908, *p* = 0.005). However, the repeated measures ANOVA did not find a significant effect of the fungal clade on the volume of the fungal gardens (F_1,11_= 0.046, *p* = 0.834). There also was not a significant interaction between the cultivar of fungus grown and the growth of the fungal garden over time (repeated measures ANOVA: F_1.32,14.56_= 0.600, *p* = 0.496). (**B**) Like *T. septentrionalis*, the volume of the fungal garden grew significantly over the course of the experiment for the *T. pomonae* subcolonies (repeated measures ANOVA: F_1.90,17.09_ = 10.048, *p* = 0.001), but there was not a significant effect of the fungal clade on the volume of the fungal gardens (repeated measures ANOVA: F_1,9_ = 0.418, *p* = 0.534). The repeated measures ANOVA did find a significant interaction between the cultivar of fungus grown and the growth of the fungal garden over time (F_1.94,17.49_ = 3.763, *p* = 0.045). Using pairwise ANOVAs, this interaction was caused by the nearly synchronous collapse of the Clade-A fungal gardens, resulting in a significant difference in the fungal garden volumes on July 28, 2021 (F_1,9_ = 28.6, *p* = 4.64e-3).

Of the twelve *T. septentrionalis* subcolonies that were provided Clade-A fungi, nine experienced symbiotic collapse between September 8, 2021 and October 6, 2021 (Figure 2A). In order to reduce the impact of seasonality on the growth of the fungal gardens, the remaining three Clade-A subcolonies were sampled on October 6, 2021 (Figure 2A). A comparison between the bacterial microbiomes of the rarefied fungal samples of those that collapsed (n = 5) and those that persisted (n = 2) found them to be similar (PERMANOVA: F = 1.36, R^2^ = 0.21, *p* = 0.29). Similarly, a comparison between the ant microbiomes of the rarefied samples of those that collapsed (n = 9) and those that persisted (n = 3) found them to be similar (PERMANOVA: F = 0.81, R^2^ = 0.07, *p* = 0.58). Given the similarity in microbiomes between Clade-A growing subcolonies that did and did not experience a collapse, that the Clade-A growing subcolonies that were not experiencing a collapse were sampled around the time of those that did collapse, and that those not experiencing a collapse were also not experiencing fungal growth, all post-donation Clade-A samples for *T. septentrionalis* were treated as in dysbiosis, as the “stable” subcolonies appear to be on the verge of symbiotic collapse (Figure 2A, Supplementary Table S1). All *T. pomonae* subcolonies provided with Clade-A fungi experienced collapse, while none of the subcolonies provided Clade-B fungi experienced collapse (Figure 2B).

### *Trachymyrmex septentrionalis *Fungus

**Figure 3:**
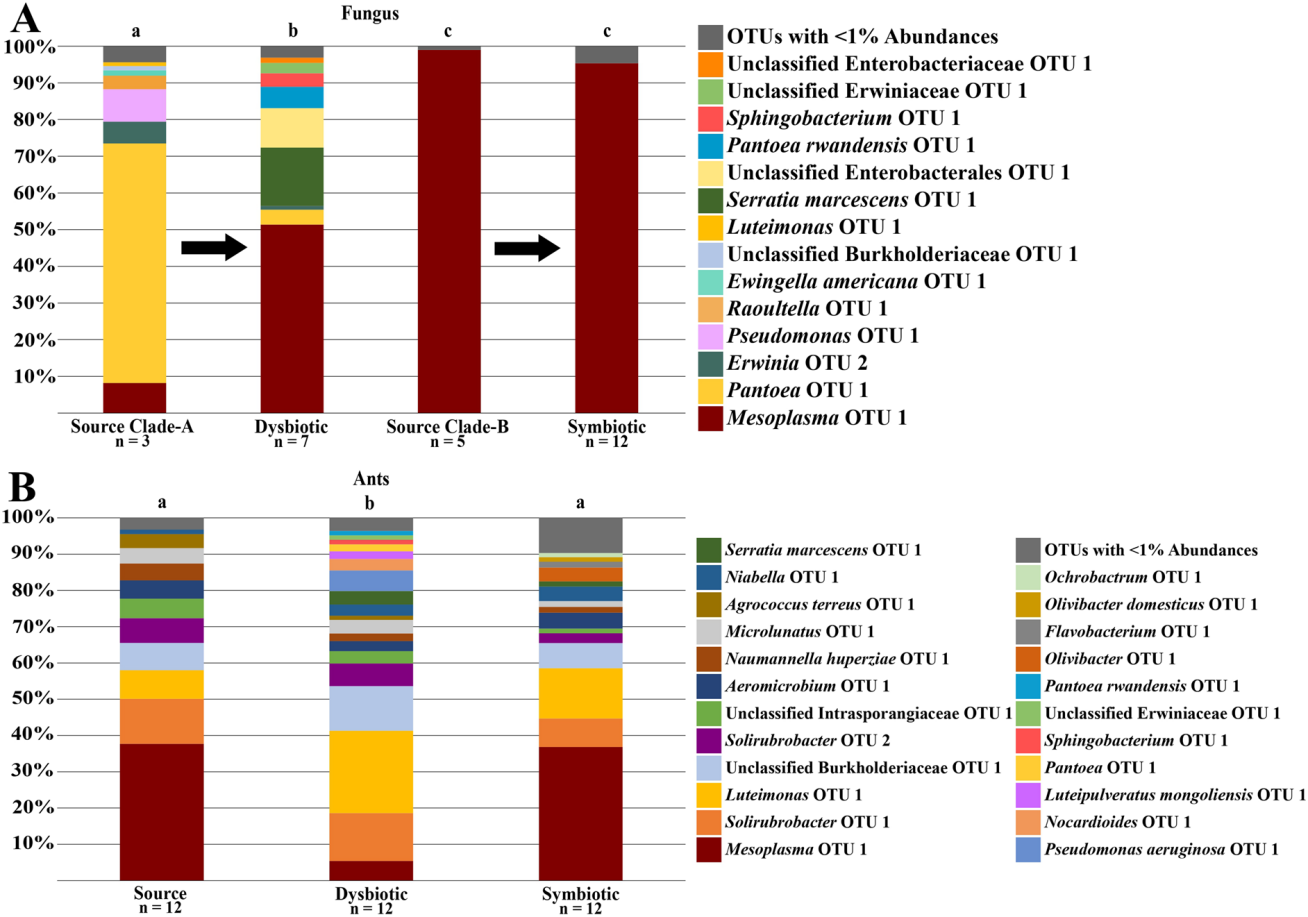
Cumulative abundance taxonomic bar plots of *Trachymyrmex septentrionalis*. Plots are for (**A**) fungus and (**B**) ant samples. Letters above the bars denote sample types that are significantly different when compared with PERMANOVA tests (*p* < 0.05). Every PERMANOVA test utilized Bray Curtis distances and had 9999 permutations. Dysbiotic fungi significantly lost *Pantoea* OTU 1 compared to their source fungi but gained *Mesoplasma* OTU 1 similar to both Clade-B fungi. Ants shared OTUs, regardless of the fungus grown, but dysbiotic ants appear to have less *Mesoplasma* OTU 1 than the other sample types.

**Figure 4:**
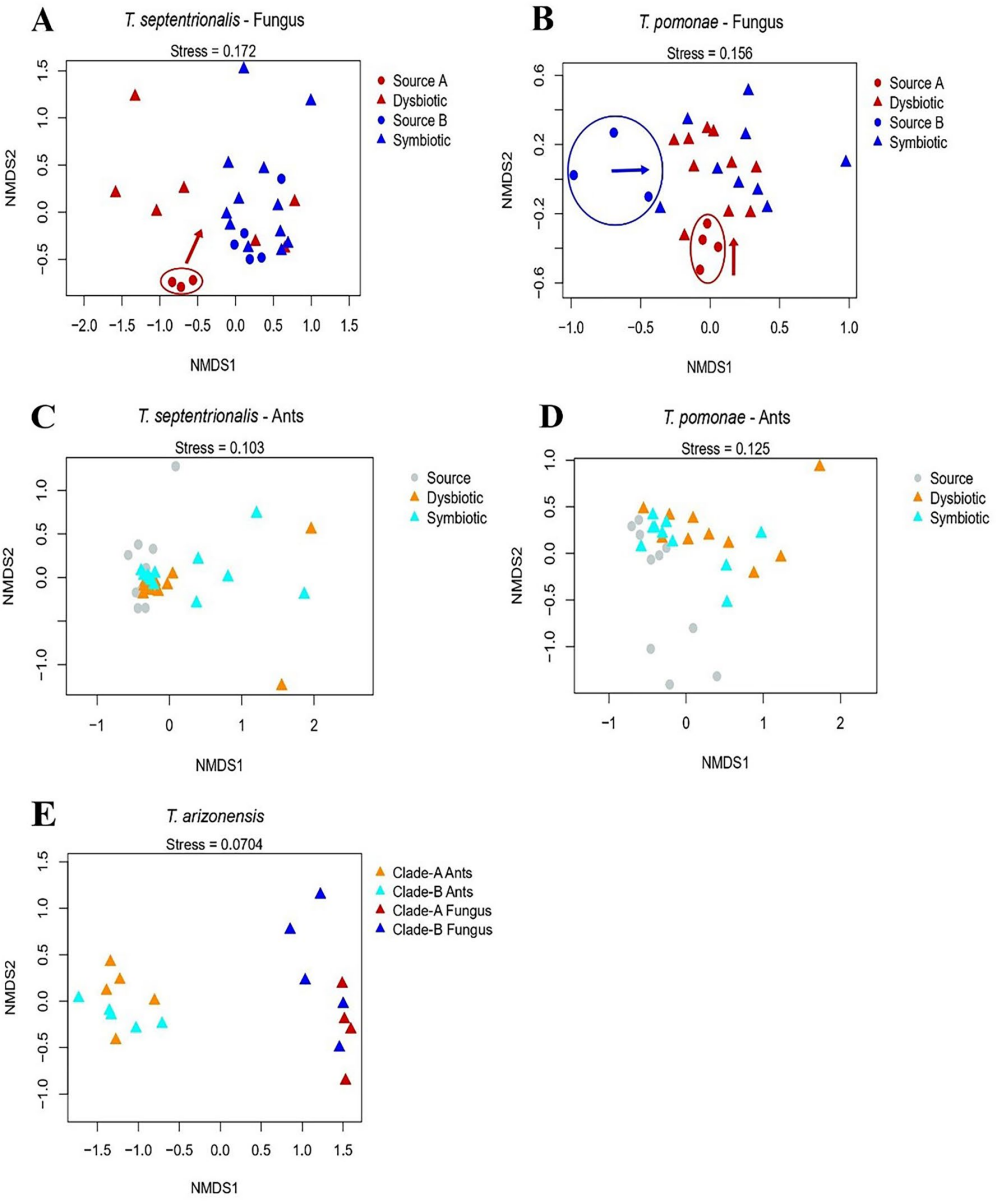
Non–metric multidimensional scaling (NMDS) plots for ant and fungal samples by species. Provided are the NMDS plots for (**A**) *T. septentrionalis* fungi, (**B**) *T. pomonae* fungi, (**C**) *T. septentrionalis* ants, (**D**) *T. pomonae* ants, and (**E**) *T. arizonensis* ants and fungi. All NMDS plots are based off Bray Curtis distances and utilize 9999 permutations. The lower the stress value associated with the NMDS plot, the better the match of the plotted distances to the community matrix distances. Arrows denote how the source fungi changed positions to their respective post-donation fungi. The *T. septentrionalis* fungi plot shows the microbiomes of dysbiotic fungi shifted to resemble symbiotic fungi more, while the *T. pomonae* fungi plot shows the source microbiomes were altered in similar ways to form the coalescent post-donation microbiomes.

The bacterial microbiomes of fungus gardens of *T. septentrionalis* subcolonies growing novel, Clade-A fungus (dysbiotic) (n = 7) were found to be significantly different than the microbiomes of source Clade-A gardens (collected from an *Atta texana* garden) (n = 3) (PERMANOVA: F = 3.71, R^2^ = 0.32, *p* = 0.017) (Figure 3A). The bacterial microbiome of dysbiotic gardens and Clade-B gardens grown by the subcolonies (symbiotic) (n = 12) were also found to be statistically different from one another (PERMANOVA: F = 5.46, R^2^ =0.24, *p* = 0.006) (Figure 3A). However, the bacterial communities of symbiotic gardens and their source Clade-B fungi (collected from a *T. septentrionalis* garden) (n = 5) were found to be not statistically different (PERMANOVA: F = 0.55, R^2^ = 0.04, *p* = 0.405) (Figure 3A). Such findings were corroborated with the NMDS plot showing that the source Clade-A fungi and all the Clade-B fungi formed two unique clusters (Figure 4A). However, while dysbiotic gardens were statistically different than the symbiotic gardens, the dysbiotic gardens overlapped with Clade-B fungal cluster (Figure 4A). These results indicate that the bacterial microbiomes of Clade-B gardens did not change throughout the experiment and were distinct from source Clade-A gardens. Additionally, collapsed, dysbiotic gardens were more similar to Clade-B gardens than their source fungal gardens.

The three most common bacterial OTUs in the microbiome of the source Clade-A gardens and dysbiotic gardens grown by *T. septentrionalis* were different. Source Clade-A gardens contained *Pantoea* OTU 1 (65.29% ± 20.05%), *Pseudomonas* OTU 1 (8.86% ± 12.53%), and *Mesoplasma* OTU 1 (8.19% ± 6.51%), whereas dysbiotic gardens contained predominately *Mesoplasma* OTU 1 (51.39% ± 41.87%), *Serratia marcescens* OTU 1 (15.88% ± 33.54%), and Unclassified Enterobacterales OTU 1 (10.76% ± 22.13%) (Figure 3A, Supplementary Table S6). Thus, *Pantoea* was largely lost and *Mesoplasma* abundance increased in dysbiotic gardens. Additionally, the dysbiotic gardens and all Clade-B gardens differed from each other due to the Clade-B gardens being primarily comprised of *Mesoplasma* OTU 1 (Source Clade-B: 99.00% ± 0.56%; Symbiotic: 95.38% ± 6.87%) (Figure 3A, Supplementary Table S6).

As the microbial communities of the source and dysbiotic Clade-A gardens grown by *T. septentrionalis* were significantly different, as were the dysbiotic and symbiotic gardens, SIMPER analyses were performed to see what OTUs were driving these differences between communities by finding which OTUs contributed the highest percentage to the total dissimilarity between the communities. Both the SIMPER analysis and ISA indicated that the low abundance of *Pantoea* OTU 1 within dysbiotic gardens was key in driving the difference between the bacterial communities of source Clade-A and dysbiotic Clade-A gardens (Table 1, Supplementary Table S7). Additionally, the SIMPER analysis found the increased abundance of *Mesoplasma* OTU 1 in the dysbiotic Clade-A gardens also caused the difference between the bacterial communities of source and dysbiotic Clade-A gardens (Table 1). This increase in *Mesoplasma* OTU 1 in the dysbiotic Clade-A gardens did not reach the high abundance of *Mesoplasma* OTU 1 found in the source Clade-B source or symbiotic Clade-B gardens, but appears to occupy an intermediary state between the symbiotic Clade-B gardens and the source Clade-A gardens.

**Table 1:** Results of similarity percentage (SIMPER) analysis for fungal samples. SIMPER results comparing sample types of *T. pomonae* and *T. septentrionalis* fungi found to be significantly different when compared using PERMANOVAs. Only OTUs with the highest contribution to cumulative dissimilarity and that together add up to 70% cumulative dissimilarity are provided for each comparison. SIMPERs were conducted using 9999 permutations. The significant differences seen in *T. septentrionalis* fungi appear to be due to the decrease in *Pantoea* OTU 1 and increase in *Mesoplasma* OTU 1 from the source Clade-A to the dysbiotic fungus, while the increase in *Mesoplasma* OTU 1 drove the difference between dysbiotic and symbiotic fungi. The significant differences seen in source Clade-A and dysbiotic *T. pomonae* fungi appear to be due to the decrease in Unclassified Rhizobiaceae OTU 1, while the increase in *Spiroplasma* OTU 1 and decrease in *Pseudonocardia* OTU 1 drove the difference between source Clade-B and symbiotic fungi.

Sample Type Comparison (Sample Type A x Sample Type B)	OTU	Average Dissimilarity Contribution (%)	Contribution to Cumulative Dissimilarity (%)	Cumulative Dissimilarity (%)	Mean Read Abundance	
					Sample Type A	Sample Type B
***T. septentrionalis***						
Source Clade-A x Dysbiotic	*Pantoea* OTU 1	30.63	34.6	34.6	457.00	28.10
	*Mesoplasma* OTU 1	22.99	26.0	60.6	57.30	359.70
	*Serratia marcescens* OTU 1	7.93	9.0	69.6	0.30	111.10
	Unclassified Enterobacterales OTU 1	5.38	6.0	75.6	0.00	75.30
Dysbiotic x Symbiotic	*Mesoplasma* OTU 1	23.51	47.1	47.1	359.70	667.70
	*Serratia marcescens* OTU 1	7.93	15.8	62.9	111.10	0.40
	Unclassified Enterobacterales OTU 1	5.38	10.8	73.7	75.30	0.00
***T. pomonae***						
Source Clade-A x Dysbiotic	Unclassified Rhizobiaceae OTU 1	42.13	48.2	48.2	364.20	46.90
	*Serratia marcescens* OTU 1	11.33	13.0	61.2	0.80	86.30
	*Spiroplasma* OTU 1	8.85	10.1	71.3	2.80	67.40
Source Clade-B x Symbiotic	*Spiroplasma* OTU 1	17.44	17.6	17.6	5.67	133.22
	*Pseudonocardia* OTU 1	12.11	12.3	29.9	92.00	0.00
	*Variovorax* OTU 1	8.64	8.7	38.6	65.67	0.00
	*Gluconobacter* OTU 1	7.73	7.8	46.4	0.00	58.78
	*Wolbachia* OTU 1	6.27	6.4	52.8	47.67	0.00
	*Stenotrophomonas* OTU 1	5.41	5.4	58.2	0.00	41.11
	*Rosenbergiella* OTU 1	5.21	5.3	63.5	0.00	39.56
	*Pantoea* OTU 1	4.62	4.7	68.2	0.00	35.11
	*Cutibacterium acnes* OTU 1	4.61	4.6	72.8	35.00	0.00

### *Trachymyrmex pomonae* Fungus

**Figure 5:**
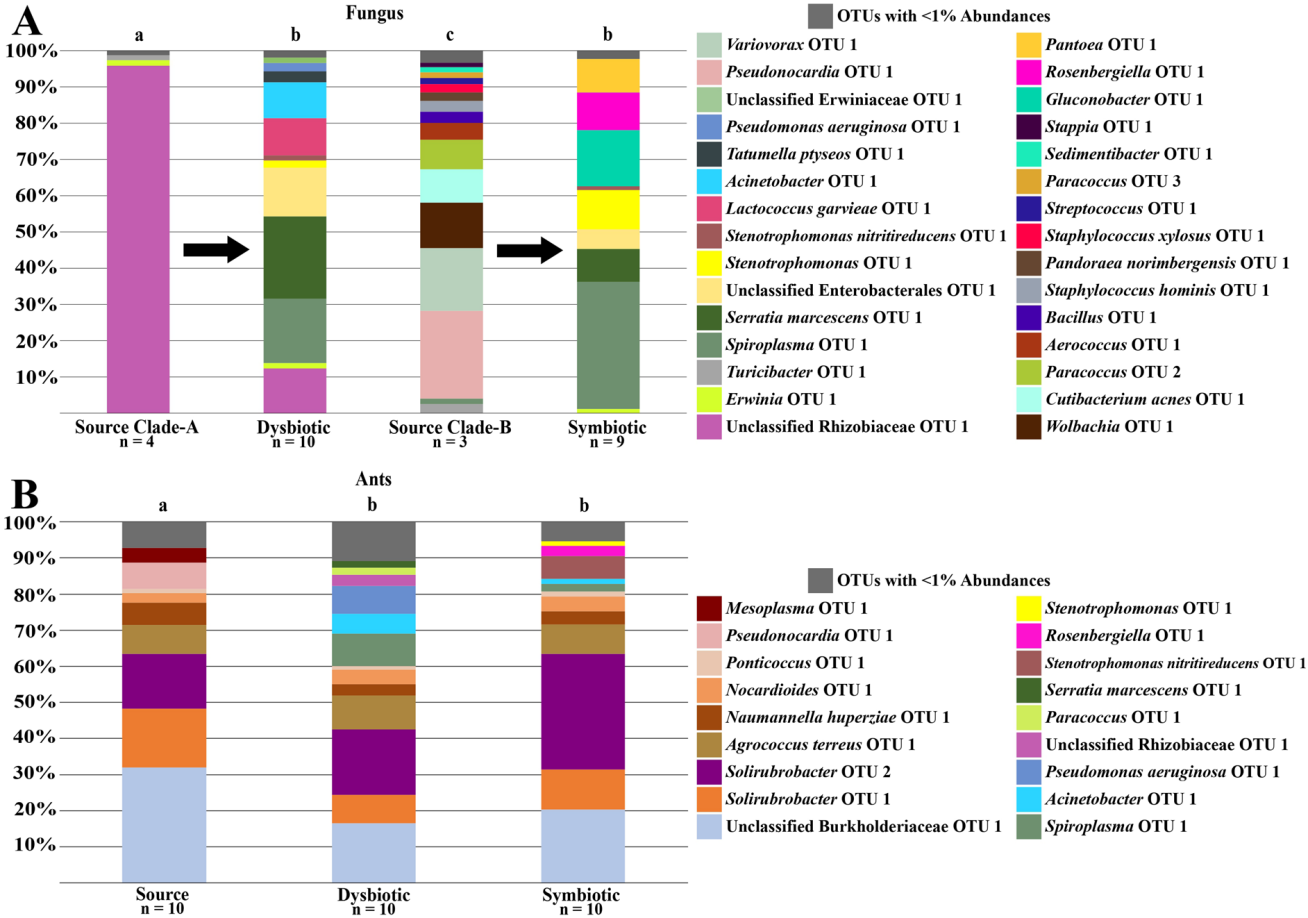
Cumulative abundance taxonomic bar plots of *Trachymyrmex pomonae*. Plots are for (**A**) fungus and (**B**) ant samples. Letters above the bars denote sample types that are significantly different when compared with PERMANOVA tests (*p* < 0.05). Every PERMANOVA test utilized Bray Curtis distances and had 9999 permutations. Dysbiotic fungi significantly lost Unclassified Rhizobiaceae OTU 1 in comparison to their source fungi, but gained *Spiroplasma* OTU 1 similar to symbiotic fungi. All ants shared OTUs but there are notable differences, such as with *Pseudonocardia* OTU 1 being present in only the source ants.

The bacterial communities of *T. pomonae* subcolonies growing novel, Clade-A gardens (dysbiotic) (n = 10) and Clade-B gardens (symbiotic) (n = 9) were not statistically different from each other (PERMANOVA: F = 1.05, R^2^ = 0.06, *p* = 0.368) (Figure 5A). However, the bacterial communities of the source Clade-A fungi (collected from an *T. arizonensis* garden) (n = 4) were significantly different from the dysbiotic Clade-A gardens (PERMANOVA: F = 4.47, R^2^ = 0.27, *p* = 0.008) and the bacterial communities of source Clade-B gardens (collected from an *T. arizonensis* garden) (n = 3) were significantly different from the symbiotic Clade-B gardens (PERMANOVA: F = 2.23, R^2^ = 0.18, *p* = 0.009) (Figure 5A). Such findings were corroborated with the NMDS plot showing that the dysbiotic and symbiotic fungi formed their own respective cohesive cluster, while the source fungi formed clade-dependent clusters (Figure 4B). These results suggest that the source microbial communities from the different fungal clades were altered from a unique, clade-dependent state, to communities that were similar, even between the two clades.

The three most common bacterial OTUs in the microbiome of the source and dysbiotic Clade-A fungus differed; with source Clade-A’s being Unclassified Rhizobiaceae OTU 1 (95.86% ± 5.70%), *Erwinia* OTU 1 (1.51% ± 2.62%), and *Turicibacter* OTU 1(1.38% ± 2.39%), whereas the dysbiotic Clade-A fungi were *Serratia marcescens* OTU 1 (22.71% ± 32.76%), *Spiroplasma* OTU 1 (17.74% ± 31.22%), and Unclassified Enterobacterales OTU 1 (13.42% ± 23.24%) (Figure 5A, Supplementary Table S6). Additionally, the three most common bacterial OTUs in the microbiome of the source and symbiotic Clade-B fungus differed; with the source Clade-B’s being *Pseudonocardia* OTU 1 (24.21% ± 26.81%), *Variovorax* OTU 1 (17.28% ± 12.39%), and *Wolbachia* OTU 1 (12.54% ± 8.90%), whereas the symbiotic Clade-B fungi were *Spiroplasma* OTU 1 (35.06% ± 45.00%), *Gluconobacter* OTU 1 (15.47% ± 32.29%), and *Stenotrophomonas* OTU 1 (10.82% ± 27.89%) (Figure 5A, Supplementary Table S6). Notably, the bacterial communities of the dysbiotic Clade-A and symbiotic Clade-B fungal gardens were similar, per the PERMANOVA tests, but the top three most abundant taxa differed between them (Figure 5A, Supplementary Table S6).

As the microbial communities of the source and dysbiotic Clade-A gardens were significantly different, as well as the source and symbiotic Clade-B gardens, SIMPER analyses were performed to see what OTUs were driving these differences in the communities by finding which OTUs contributed the highest percentage to the total dissimilarity between the communities. Both the SIMPER analysis and ISA found the loss of Unclassified Rhizobiaceae OTU 1 in dysbiotic Clade-A gardens was the driving cause for the difference in the bacterial community between source and dysbiotic Clade-A gardens (Table 1, Supplementary Table S7). The SIMPER analysis found the loss of *Pseudonocardia* OTU 1 in symbiotic Clade-B gardens was the driving cause for the difference in the bacterial community between source and symbiotic Clade-B gardens (Table 1). Additionally, SIMPER analyses found the increase in abundance of *Spiroplasma* OTU 1 for both dysbiotic Clade-A and symbiotic Clade-B gardens appeared to aid in the significant differences between them and their respective source fungi (Table 1).

### *Trachymyrmex septentrionalis* and *Trachymyrmex pomonae* Ants

*T. septentrionalis* ants in subcolonies growing Clade-A fungus (dysbiotic) (n = 12) were found to have different microbiomes than those of ants in subcolonies growing Clade-B fungi (symbiotic) (n = 12) and were different than ants acting as the sources to the subcolonies (n = 12) (Figure 3B, Figure 4C, Supplementary Table S8). The three most common bacterial OTUs in the microbiomes of the three ant sample types were similar (Figure 3B, Supplementary Table S9). The SIMPER analyses and the ISAs suggests that the primary difference between the bacterial communities of ants growing Clade-A fungi and the source ants, as well as the ants growing Clade-B fungi, was the lower abundance of *Mesoplasma* OTU 1 and the higher abundance of *Luteimonas* OTU 1 within the ants growing Clade-A fungi (Table 2, Supplementary Table S10).

**Table 2:** Results of similarity percentage (SIMPER) analysis for ant samples. SIMPER results comparing sample types of *T. pomonae* and *T. septentrionalis* ants found to be significantly different when compared using PERMANOVAs. Only OTUs with the highest contribution to cumulative dissimilarity and that together add up to 70% cumulative dissimilarity are provided for each comparison. SIMPERs were conducted using 9999 permutations. The significant differences seen in *T. septentrionalis* ants appear to be due to the low abundance of *Mesoplasma* OTU 1 and the presence of *Luteimonas* OTU 1 in dysbiotic ants. The significant differences seen in *T. pomonae* ants appear to be due to differences in core taxa (such as Unclassified Burkholderiaceae OTU 1 and *Solirubrobacter* OTU 2) and the presence of lower abundance, sample-type specific OTUs (such as *Acinetobacter* OTU 1 in dysbiotic ants and *Stenotrophomonas nitritireducens* OTU 1 in symbiotic ants).

Sample Type Comparison (Sample Type A x Sample Type B)	OTU	Average Dissimilarity Contribution (%)	Contribution to Cumulative Dissimilarity (%)	Cumulative Dissimilarity (%)	Mean Read Abundance	
					Sample Type A	Sample Type B
***T. septentrionalis***						
Source x Dysbiotic	*Mesoplasma* OTU 1	18.31	28.6	28.6	263.83	37.67
	*Luteimonas* OTU 1	9.43	14.7	43.3	55.08	158.92
	*Solirubrobacter* OTU 1	4.76	7.4	50.7	87.00	92.67
	Unclassified Burkholderiaceae OTU 1	4.10	6.4	57.1	52.58	86.08
	*Pseudomonas aeruginosa* OTU 1	2.83	4.4	61.5	0.00	39.67
	*Solirubrobacter* OTU 2	2.75	4.3	65.8	47.83	43.67
	*Aeromicrobium* OTU 1	2.59	4.1	69.9	35.25	19.33
	Unclassified Intrasporangiaceae OTU 1	2.59	4.0	73.9	37.67	23.75
Dysbiotic x Symbiotic	*Mesoplasma* OTU 1	16.62	26.4	26.4	37.67	257.83
	*Luteimonas* OTU 1	8.22	13.0	39.4	158.92	96.83
	*Solirubrobacter* OTU 1	4.66	7.4	46.8	92.67	55.17
	Unclassified Burkholderiaceae OTU 1	4.02	6.3	53.1	86.08	48.42
	*Pseudomonas aeruginosa* OTU 1	2.83	4.5	57.6	39.67	0.08
	*Solirubrobacter* OTU 2	2.33	3.7	61.3	43.67	19.00
	*Niabella* OTU 1	2.24	3.6	64.9	21.58	28.00
	*Serratia marcescens* OTU 1	2.12	3.3	68.2	26.08	10.08
	*Olivibacter* OTU 1	1.96	3.2	71.4	1.25	26.67
***T. pomonae***						
Source x Dysbiotic	Unclassified Burkholderiaceae OTU 1	10.43	17.7	17.7	121.50	63.10
	*Solirubrobacter* OTU 2	5.65	9.5	27.2	57.20	68.80
	*Solirubrobacter* OTU 1	5.25	8.9	36.1	62.20	29.70
	*Spiroplasma* OTU 1	4.59	7.8	43.9	1.40	34.10
	*Agrococcus terreus* OTU 1	4.14	7.0	50.9	30.70	35.90
	*Pseudomonas aeruginosa* OTU 1	3.86	6.5	57.4	0.00	29.30
	*Pseudonocardia* OTU 1	3.63	6.2	63.6	27.80	0.90
	*Naumannella huperziae* OTU 1	2.86	4.8	68.4	23.20	11.40
	*Acinetobacter* OTU 1	2.84	4.8	73.2	3.10	20.60
Source x Symbiotic	*Solirubrobacter* OTU 2	10.12	19.6	19.6	57.20	121.70
	Unclassified Burkholderiaceae OTU 1	10.00	19.4	39.0	121.50	77.40
	*Solirubrobacter* OTU 1	4.66	9.0	48.0	62.20	42.10
	*Pseudonocardia* OTU 1	3.62	7.0	55.0	27.80	0.80
	*Agrococcus terreus* OTU 1	3.40	6.6	61.6	30.70	30.60
	*Stenotrophomonas nitritireducens* OTU 1	3.18	6.2	67.8	0.00	24.20
	*Naumannella huperziae* OTU 1	2.94	5.7	73.5	23.30	14.20

The bacterial communities of *T. pomonae* ants growing Clade-A fungus (dysbiotic) (n = 10) and those growing Clade-B gardens (symbiotic) (n = 10) were not statistically different from one another, but were both significantly different than the ants acting as the sources to the subcolonies (n = 10) (Figure 4D, Figure 5B, Supplementary Table S8). Like *T. septentrionalis*, the three most common bacterial OTUs in the microbiomes of the three ant sample types were similar (Figure 5B, Supplementary Table S9). The SIMPER analysis and the ISA yielded conflicting results as to what taxa were causing these differences, with the ISA mainly indicating OTUs that were present only in their respective sample types whereas the SIMPER analysis highlighted variations in shared taxa among sample types (Table 2, Supplementary Table S10).

### *Trachymyrmex arizonensis*

**Figure 6:**
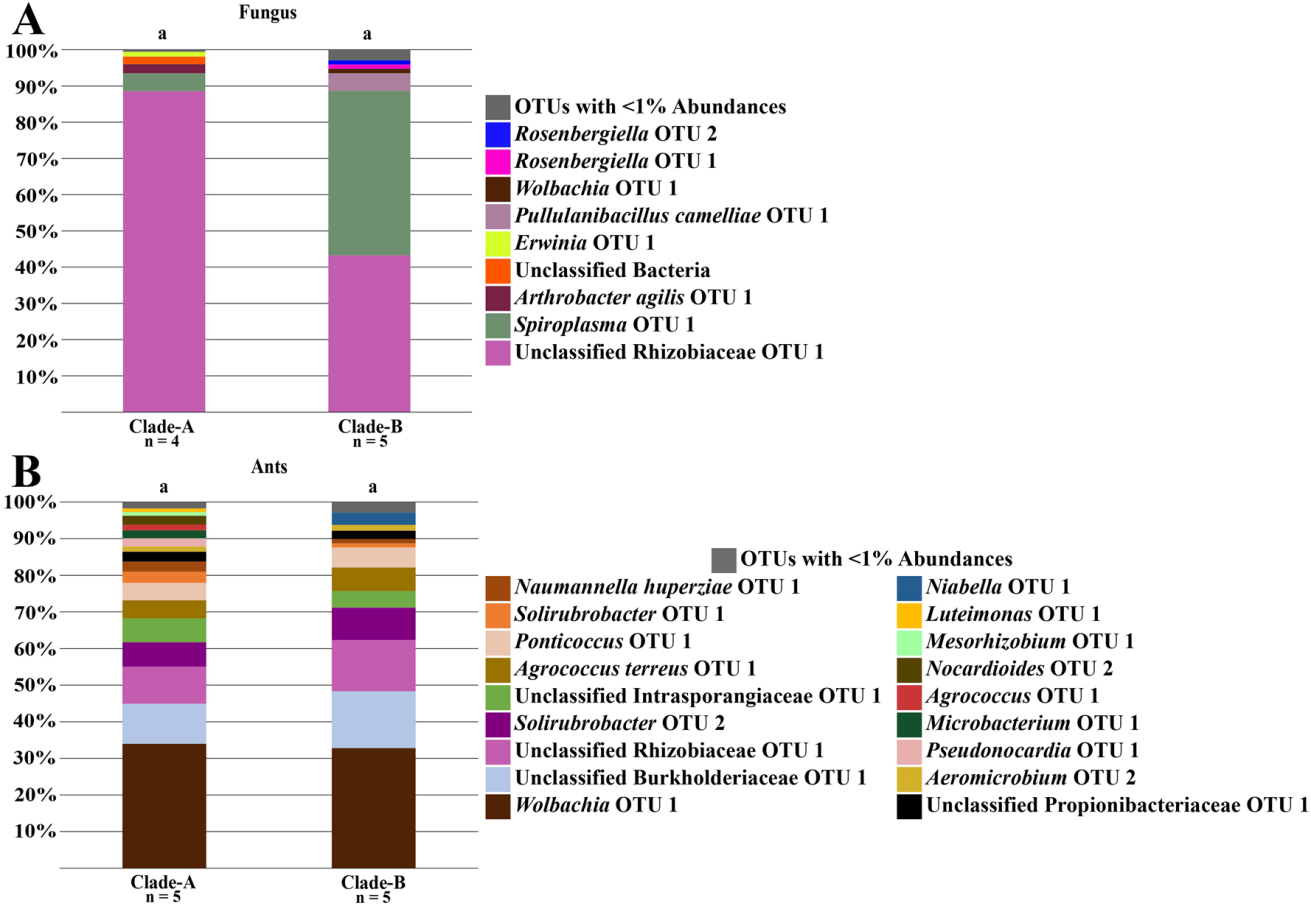
Cumulative abundance taxonomic bar plots of *Trachymyrmex arizonensis*. Plots are for (**A**) fungus and (**B**) ant samples. Letters above the bars denote sample types that are significantly different when compared with PERMANOVA tests (*p* < 0.05). Every PERMANOVA test utilized Bray Curtis distances and had 9,999 permutations. *T. arizonensis* fungi appeared to differ, with Clade-B fungi containing a higher proportion of *Spiroplasma* OTU 1 at the expense of Unclassified Rhizobiaceae OTU 1. *T. arizonensis* ants have similar bacterial taxonomic profiles, regardless of the fungus grown.

The bacterial communities of Clade-A fungal gardens (n = 4) were not significantly different from the Clade-B gardens (n = 5) (PERMANOVA: F = 3.63, R^2^ = 0.34, *p* = 0.107) (Figure 6A). Similarly, the microbiomes of ants growing native, Clade-A fungi (n = 5) were not significantly different from those growing Clade-B fungi (n = 5) (PERMANOVA: F = 0.10, R^2^ = 0.01, *p* = 0.961) (Figure 6B). These results suggest that the bacterial communities of the ants and fungi were similar regardless of the fungal clade, with the fungus gardens showing more of a difference than the ants. Such results were also corroborated with the NMDS plot showing higher overlap between the bacterial community compositions of the ants than between the Clade-A and Clade-B fungi (Figure 4E).

The three most common bacterial OTUs in the microbiome of *T. arizonensis* ants growing either Clade-A or Clade-B were the same, and included *Wolbachia* OTU 1 (Clade-A: 33.96% ± 33.18%; Clade-B: 32.78% ± 26.70%), Unclassified Burkholderiaceae OTU 1 (Clade-A: 10.94% ± 5.78%; Clade-B: 15.50% ± 9.13%), and Unclassified Rhizobiaceae OTU 1 (Clade-A: 10.12% ± 18.08%; Clade-B: 14.00% ± 27.11%) (Figure 6B, Supplementary Table S9). The three most common bacterial OTUs of Clade-A gardens were Unclassified Rhizobiaceae OTU 1 (88.60% ± 11.95%), *Spiroplasma* OTU 1 (4.95% ± 8.57%), and *Arthrobacter agilis* OTU 1 (2.5% ± 4.33%) (Figure 6A, Supplementary Table S6), whereas the three most common bacterial OTUs in Clade-B gardens were *Spiroplasma* OTU 1 (45.38% ± 37.25%), Unclassified Rhizobiaceae OTU 1 (43.24% ± 32.18%), and *Pullulanibacillus camelliae* OTU 1 (4.92% ± 8.64%) (Figure 6A, Supplementary Table S6). Thus *Spiroplasma* OTU 1 was more abundant in Clade-B gardens than Clade-A gardens, whereas Unclassified Rhizobiaceae OTU 1 was more abundant in Clade-A gardens than Clade-B gardens.

As SIMPER analyses provide taxa to describe the differences between groupings, regardless of whether or not the groups are significantly different, none were performed between the *T. arizonensis* ants or fungi samples as they were found to be similar in the PERMANOVA tests. The ISA on the Clade-A and Clade-B ants found no indicator taxa that differentiated the ants (Supplementary Table S10). Additionally, the ISA on the Clade-A and Clade-B fungi found no indicator taxa that differentiated the fungus (Supplementary Table S7).

## Discussion

The goal of this study was to compare the impact of horizontal exchange on the bacterial microbiome structure of ants and fungus garden combinations that were known to be symbiotic and dysbiotic. If the structure of the bacterial microbiome was independent of the host-symbiont combinations, there should have been no changes or random changes as a result of cross-fostering experiments. On the other hand, if the bacterial microbiome has a role in the maintenance of stability or causes dysbiosis, there should have been clear changes in combinations known to result in dysbiosis. We report the latter—dysbiotic associations in the two species studied show similar and perhaps predictable responses. The most striking discovery in our experiments was that ants provided with novel fungi converted the existing microbiome into a structure similar to that found while growing a native fungal symbiont and that this new configuration of microbiome is associated with instability and the collapse of the symbiosis. It appears that ant-fungal specificity and stability is conferred by ants actively manipulating bacterial microbiomes in ways that are synergistic with their native type of fungus. As a result, the microbiome is likely adaptive and involved in symbiotic homeostasis.

The strongest evidence that ants are sculpting their garden microbiome came from the finding that collapsing Clade-A gardens of *T. pomonae* and *T. septentrionalis* contained bacterial microbiomes close to those of healthy Clade-B gardens grown by these species than of the original structure of their provided Clade-A gardens. As Clade-A gardens collapse when experiencing these species-specific microbiome communities, there appears to be some incompatibility between Clade-A fungi and the microbial communities these ants are constructing. Additionally, as these patterns were observed in both *T. pomonae* and *T. septentrionalis,* they were unlikely the outcome of slightly different methods of subcolony establishment in these two species. On the other hand, *T. arizonensis* microbial communities from long-term, stable, laboratory colonies were distinct from the other two ant species but did not significantly change when growing the different fungal clades. As a result, their particular microbial configuration is stable when growing either type of fungus.

One hypothetical explanation is that stable associations are characterized by a highly interactive microbial community and dysbiosis may result when horizontal exchange disrupts the interactions in these communities. For example, the main taxa that appear to be manipulated by *T. pomonae* and *T. septentrionalis* are involved in nitrogen cycling. In both *T. pomonae* and *T. septentrionalis*, Mollicutes (*Mesoplasma* OTU 1 and *Spiroplasma* OTU 1) were found to increase in abundance whereas Unclassified Rhizobiaceae OTU 1 and *Pantoea* OTU 1 were found to decrease in collapsing Clade-A gardens. *Pantoea* has been previously found to fix nitrogen within the fungal garden and members of Rhizobiales are suspected of fixing nitrogen within the gut of *Acromyrmex* ants^31,32^. Attine associated Mollicutes have also been found to convert arginine into NH_3_ within the gut of the ants^33^. As these taxa are all suspected of fixing nitrogen, Mollicute taxa within the microbiomes of the fungi would initially appear to be interchangeable and redundant if another nitrogen fixing bacteria is present. However, as Clade-A fungal gardens collapse under these altered microbial communities, specificity between nitrogen fixing bacteria and the fungus garden must be present in natural Clade-B gardens when grown by *T. pomonae* or *T. septentrionalis*.

Although the bacterial microbiomes of fungus gardens grown by *T. arizonensis* were not significantly different, the *p*-values were low enough for a Type II error to be a concern. As a result, similar mechanisms of microbiome structuring may be involved in *T. arizonensis* symbioses. For example, even though the gardens of *T. arizonensis* were composed of nitrogen fixers and Mollicutes, gardens were nearly split 50:50 between these two taxa in Clade-B gardens. Clade-A fungus grown by *T. arizonensis* was primarily comprised of Unclassified Rhizobiaceae OTU 1, with reduced relative abundance of Mollicutes (Figure 6A). It seems possible that *T. arizonensis* is able to prevent an overabundance of Mollicutes, which may be lethal for Clade-A fungi, which is a hypothesis raised by Meirelles *et al*. (2016). As *T. pomonae* and *T. septentrionalis* are phylogenetically closer related to each other than with *T. arizonensis* who also shares a clade with other *Trachymyrmex* species capable of growing Clade-A fungi (e.g. *T. desertorum*), the mechanisms that cause lower concentrations of Mollicutes may have evolved in this separate clade^39^. Future studies looking at the metabolomics of these Clade-A nitrogen fixing bacteria are required to see if they supply the fungus additional enzymes or secondary compounds.

The significant variation seen with the ants were primarily driven by core OTUs found across all sample types, such as the *Solirubrobacter* OTUs and Unclassified Burkholderiaceae OTU 1, as well as low abundance OTUs that were likely environmentally acquired, such as *Pseudomonas aeruginosa* OTU 1 and *Acinetobacter* OTU 1. Both *Solirubrobacter* and members of Burkholderiaceae have been commonly found in *T. septentrionalis* ant microbiomes^34–36^. Aside from fungus-gardening ants, *Solirubrobacter* has been found in soil crusts and earthworm burrows, and is suspected of aiding in the growth of certain plants^65,66^. The consistency of finding *Solirubrobacter* on *T. septentrionalis* and *T. pomonae* ants, even within laboratory settings, suggests these bacteria form tight symbiotic relationships with the ants and are not strictly a transient artifact of a subterranean lifestyle^34–36^. Notably, *Pseudonocardia*, a heavily studied bacteria within the attine symbiosis, appears to have been lost during the experiment for both dysbiotic and symbiotic *T. pomonae* ants. Inhibitory interactions between common antibiotic producing attine-derived bacteria can occur, as seen with plated strains of *Amycolatopsis* found to inhibit the growth of *Pseudonocardia*^30^. Future quantitative sequencing approaches may examine whether the dynamics of altered microbiomes in ants growing Clade-A fungi create an environment where *Pseudonocardia* are outcompeted by other taxa.

While the mechanisms that determine symbiosis/ dysbiosis in fungus-gardening ant symbiosis appear to be complex, it is clear that when ant-fungal combinations fail, bacterial communities also disassociate and that the disassociation is not random. As a result, future work needs to take an explicitly community ecology approach and examine the interactions among bacteria, ants, and fungi at a very fine scale^67^. In particular, as it appears that nitrogen cycling is impacted by relative changes in Mollicutes, Rhizobiales, and *Pantoea* abundance, future work may examine how these changes are actually determined (if at all) by host biology or the presence or absence by other taxa, such as Actinobacteria or *Solirubrobacter*. Future work will need to additionally examine the interactions among hosts and bacteria with other members of the symbiotic community (such as microfungi and yeasts), many of which are known to interact with bacteria, host ants, and fungi^27,68–70^.

### Supplementary Information

Supplementary Information.

## Data Availability

Raw microbiome sequences for all samples have been uploaded to NCBI under the BioProject PRJNA 982270. Our Qiime2 pipeline, along with sample R script used during the post-processing analysis, can be found at https://github.com/bsbringhurst/Consequences-of-Horizontal-Fungus-Exchange. The resulting OTU table output by our Qiime2 pipeline can be found in the DRYAD Digital Repository (10.5061/dryad.3ffbg79pw).
