# Supplementary material for: Symbiosis, dysbiosis and the impact of horizontal exchange on bacterial microbiomes in higher fungus-gardening ants
Source: Sci Rep. 2024 Feb 8;14:3231. doi: 10.1038/s41598-024-53218-6 (PMC10853281; doi:10.1038/s41598-024-53218-6)
Supplement: Supplementary file 1 — Supplementary Information. [file 41598_2024_53218_MOESM1_ESM.pdf]

Supplementary Methods

Donor Fungus Sources

The source Clade-A fungi for *T. arizonensis* was originally from a laboratory colony of *Acromyrmex versicolor* collected in Tucson, Arizona (approximately 32°19′ N, 110°54′ W) in 2009 [1], but had since been grown by a *T. arizonensis* colony. This source of Clade-A fungus was chosen for *T. arizonensis* due to *Acromyrmex versicolor* being a sympatric species that grows Clade-A fungi, making this source of fungi a potential means for *T. arizonensis* to encounter Clade-A fungus naturally [2,3]. The source Clade-B fungi for *T. arizonensis* was donated from Clade-B fungus obtained from a mature laboratory colony of *T. arizonensis* collected at the Southwestern Research Station in 2010 [1].

The source Clade-A fungi for *T. pomonae* came from a mature *T. arizonensis* laboratory colony that was growing fungus obtained from an *Acromyrmex versicolor* colony, while the source Clade-A fungi for *T. septentrionalis* came from an *Atta texana* colony collected near Tyler, Texas on April 22, 2021. This source of Clade-A fungus was chosen for *T. pomonae* due to *Acromyrmex versicolor* and *T. arizonensis* being the only co-occurring ants growing Clade-A fungus, while the source Clade-A fungus utilized for *T. septentrionalis* was chosen due to *Atta texana* being the only co-occurring ant species growing Clade-A fungus, making these sources of fungi the only means for these species to potentially encounter Clade-A fungus naturally [2–4]. The source Clade-B fungi for *T. pomonae* was donated from Clade-B4 fungus obtained from a mature laboratory colony of *T. arizonensis,* while the source Clade-B fungi for *T. septentrionalis* was donated from Clade-B4 fungus obtained from a colony of *T. septentrionalis* collected on the University of Texas at Tyler campus on April 5, 2021.

Subcolony Care

Each subcolony's nesting dish consisted of one smaller diameter, lidless, 9 mm tall Petri dish in a larger, 15 mm tall, non–vented Petri dish. A layer of moistened dental plaster was poured into the larger petri dish to keep the smaller petri dish in place and to act as a moisture reservoir for the ants. Subcolonies were visually monitored daily. The workers were provided fresh polenta and frass from eastern tent caterpillars (*Malacosoma americanum*) and forest tent caterpillars (*Malacosoma disstria*) every other day to allow the ants to collect these substrates *ad libitum* for their fungus gardens. We did not monitor how much of the fungal substrate was consumed or the percentages of food provided, but all subcolonies received the same amount of food and the same type of food during each feeding day. Nest dishes were cleaned and maintained every other day to remove any unused substrate from the previous provision. Subcolonies were kept in saturated air at a temperature of 24–25 °C within an environmental chamber (VWR low temperature incubator, model number 10753-894), which are thought be ideal growing conditions for these ants [5]. Prior to the fungal donation, the subcolonies were fed a 10% sucrose solution diet for a week to allow the workers to purge themselves of any residual fungi and acclimate to the new nest dishes [6]. The 10% sucrose solution was replaced every two days. After this week, the ants received their fungal donation.

Fungus Clade Confirmation

To validate whether each colony/subcolony was growing the intended fungal cultivar it was experimentally given, the DNA of the resulting fungus garden grown by each subcolony was extracted, sequenced, and compared to reference sequences. The fungal DNA was extracted from the gongylidia, or the nutrient filled hyphal tips of the fungus used as the food source for the ants [7,8]. To do this, a snip of gongylidia from the fungal garden was removed using ethanol–flame

sterile techniques and placed into a 20% Chelex (Bio-Rad) solution prepared with 40uL of Chelex beads and 160 uL ddH_2O. The resulting solutions were then vortexed, centrifuged, and heated in a thermocycler (first set at 60 °C for 90 minutes, then set at 99 °C for 15 minutes). The resulting supernatant was then used to make 1:10 dilutions of supernatant:ddH_2O for the following PCRs. Genotype determination was used by amplifying a fragment of the ribosomal gene internal transcribed spacer (ITS) region [9]. The forward primer of this fragment was ITS5 (5' – GGAAGTAAAAGTCGTAACAAGG – 3') and the reverse primer was ITS4 (5' – TCCTCCGCTTATTGATATGC – 3') [10]. The PCR for the ITS fragment had the following steps: 1) have a starting temperature of 94°C for two minutes, 2) denature the solution for 35 cycles at 94°C for 1 minute, anneal the solution at 51.5°C for 1 minute, and elongate the strands at 72°C for two minutes, then 3) hold at 72°C for five minutes. The resulting PCR products were sent to the DNA Sequencing Facility at the University of Texas at Austin for Sanger sequencing on an Applied Biosystems 3730 DNA Analyzer. The resulting ITS sequences were cleaned up and aligned in Geneious 10.1.2 [11]. Sequencing errors or misreads in the DNA sequences were manually corrected. Sequences were identified using BLAST at NCBI GenBank and personal databases of ITS sequences [12].

Microbiome Sampling Details

Samples of a single worker from the collected *T. septentrionalis* and *T. pomonae* colonies were used as the source ants. Source samples of workers from each *T. septentrionalis* colony were taken three weeks after the subcolonies received their fungus donations. This delay was done to allow the microbiome of the ants to adjust to lab conditions and reduce the impact of transient environmental taxa between source and switch comparisons [13]. As the *T. pomonae* colonies were collected in 2019 and had their microbiomes adjusted to the laboratory setting,

their source samples were taken at the inception of the study. Five samples of small fungus trimmings from the fungal gardens serving as the cultivar donation sources were collected at the inception of the experiment and act as the source fungus samples.

Samples of a single worker and small trimming of fungus from *T. septentrionalis* and *T. pomonae* subcolonies were taken monthly to ensure there was a sample prior to any unforeseen total collapse of the fungal gardens. At the time of a collapse, a single worker and small trimming of fungus were sampled for microbiome analysis, with all remaining ants and healthy fungus put in vials of 100% ethanol for future potential analyses. At the point where all Clade-A subcolonies collapsed, the remaining Clade-B subcolonies had a single worker and small trimming of fungus sampled for microbiome analysis, with all remaining ants and healthy fungus put in vials of 100% ethanol for future potential analyses. These final samples act as the post-donation samples for the ants and fungus. As no unforeseen total collapses occurred, all post-donation ant and fungus samples occurred utilizing the latter methods.

Bacterial Distribution

After the raw sequences were processed and filtered through Qiime2, the dataset consisted of 1,559,132 sequences, with 887,847 sequences associated with the 77 ant samples and 671,285 sequences associated with the 75 fungal samples. Rarefying the samples per the rarefaction thresholds reduced this down to 84,380 sequences, with 46,600 sequences associated with 76 ant samples and 37,780 sequences associated with 62 fungal samples. From these rarefied samples, 218 OTUs were detected, with 161 of these OTUs found within the ant samples and 117 OTUs found within the fungal samples.

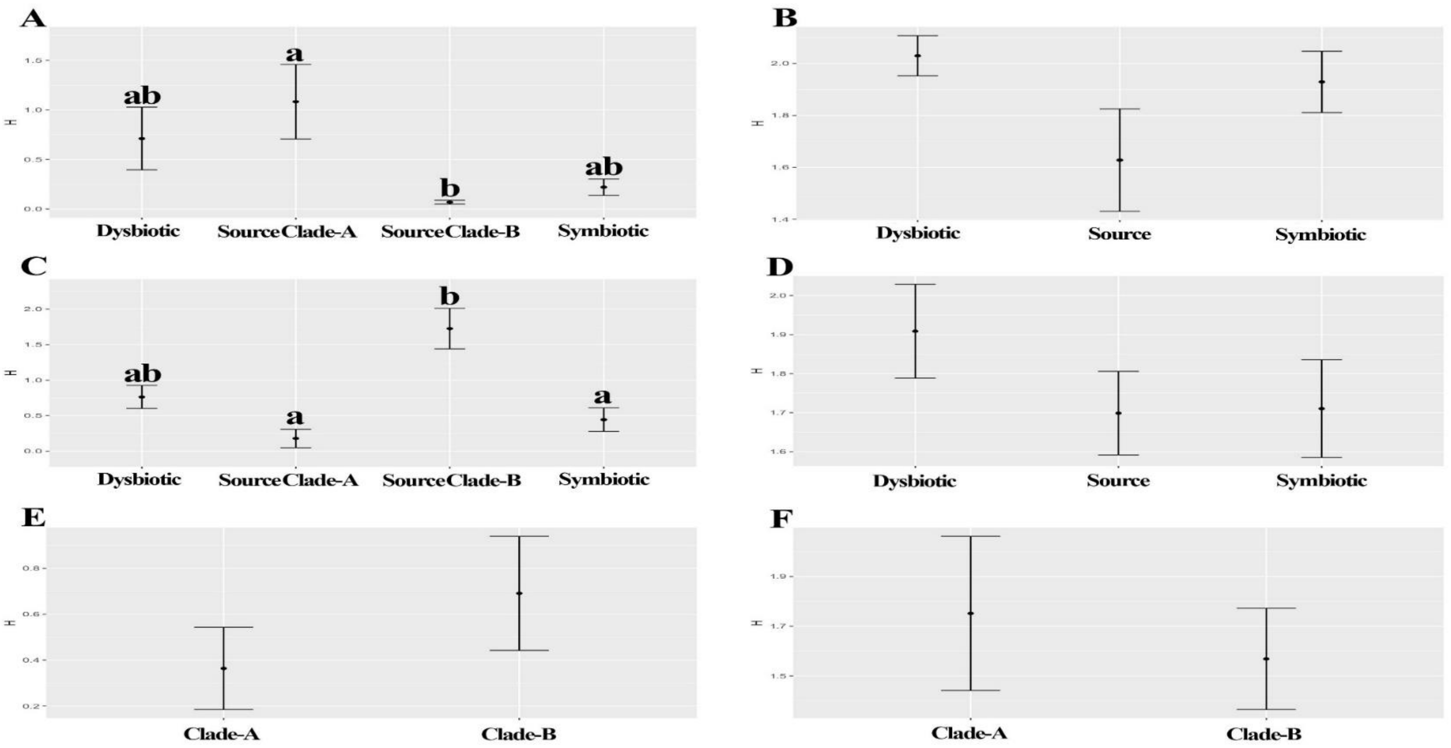

Figure S1. Shannon's diversity index (H) plots (A) *Trachymyrmex septentrionalis* fungal gardens, (B) *T. septentrionalis* ants, (C) *T. pomonae* fungal gardens, (D) *T. pomonae* ants, (E) *T. arizonensis* fungal gardens, and (F) *T. arizonensis* ants. All sample types were compared using Kruskal-Wallis tests to test for significant differences. Sample types containing different letters were found to be significantly different (Bonferroni adjusted p-value < 0.05) when tested with a Dunn's test (post-hoc test). Error bars denote the standard error around the mean. The mean Shannon's diversity indexes between the *T. septentrionalis* fungal gardens were significantly different (Kruskal-Wallis: $\chi^2 = 9.6797$, df = 3, p = 0.02149), which was driven by source Clade-B fungi having a significantly lower Shannon's index than source Clade-A fungi (Dunn's: Z = 2.956, padj = 0.019). The mean Shannon's diversity indexes between the *T. pomonae* fungal gardens were significantly different (Kruskal-Wallis: $\chi^2 = 10.357$, df = 3, p = 0.01577), which was driven by source Clade-B fungi having a more diverse community of OTUs than that of the source Clade-A fungi (Dunn's: Z = -2.869, padj = 0.025) and that of the symbiotic fungi (Dunn's: Z = 2.638, padj = 0.050).

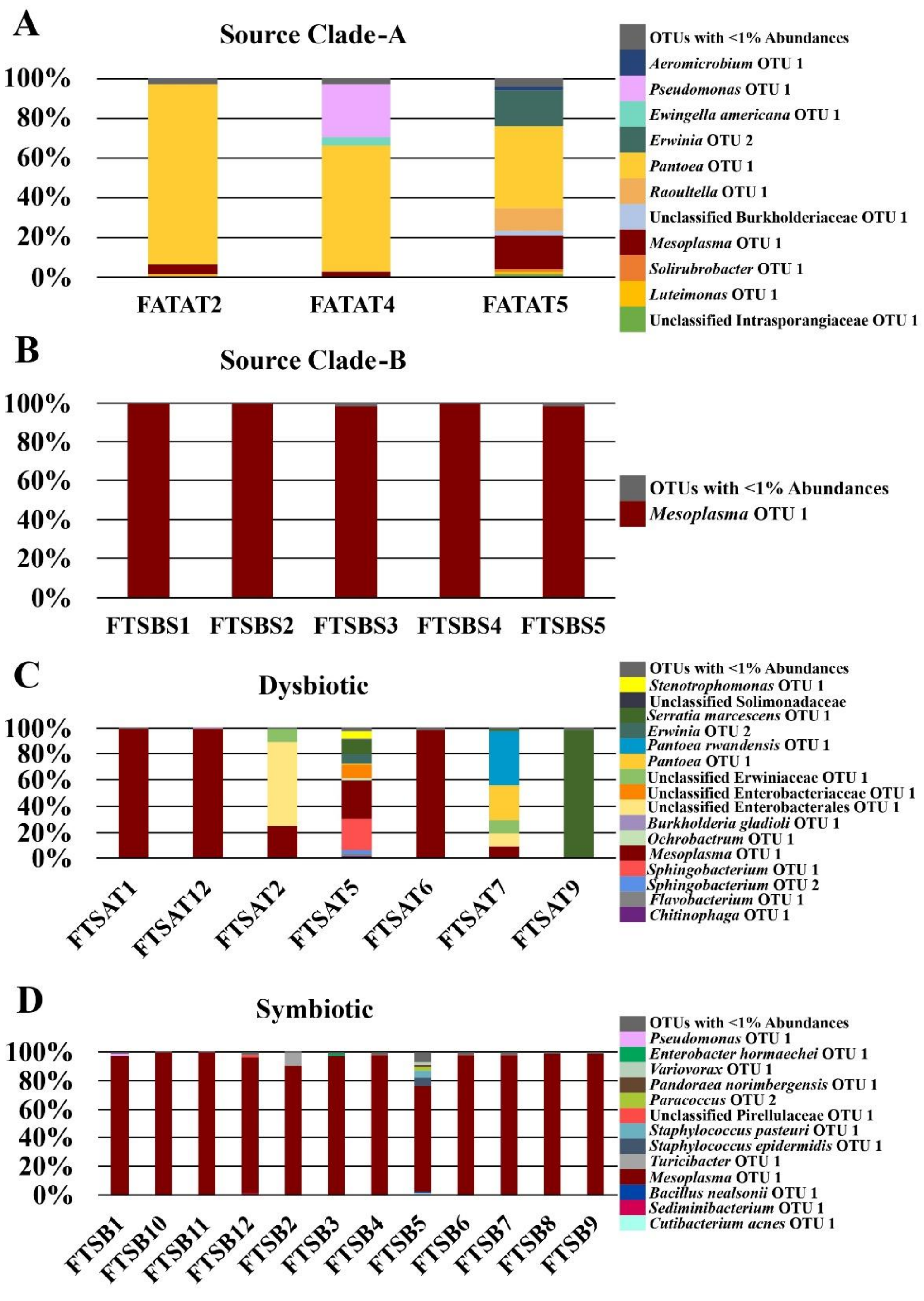

Figure S2. Taxonomic bar plots for each rarified bacteria microbiome sample of fungi associated with *Trachymyrmex septentrionalis* subcolonies and their fungal sources Taxonomic bar plots for each rarified sample of *Trachymyrmex septentrionalis* (A) source Clade-A fungi (n = 3), (B) source Clade-B fungi (n = 5), (C) dysbiotic, Clade-A fungi (n = 7), and (D) symbiotic, Clade-B fungi (n = 12). Bar plots are based on OTUs that occur in abundances greater than 1%. Sample identifiers correspond to those in Supplemental Table S1.

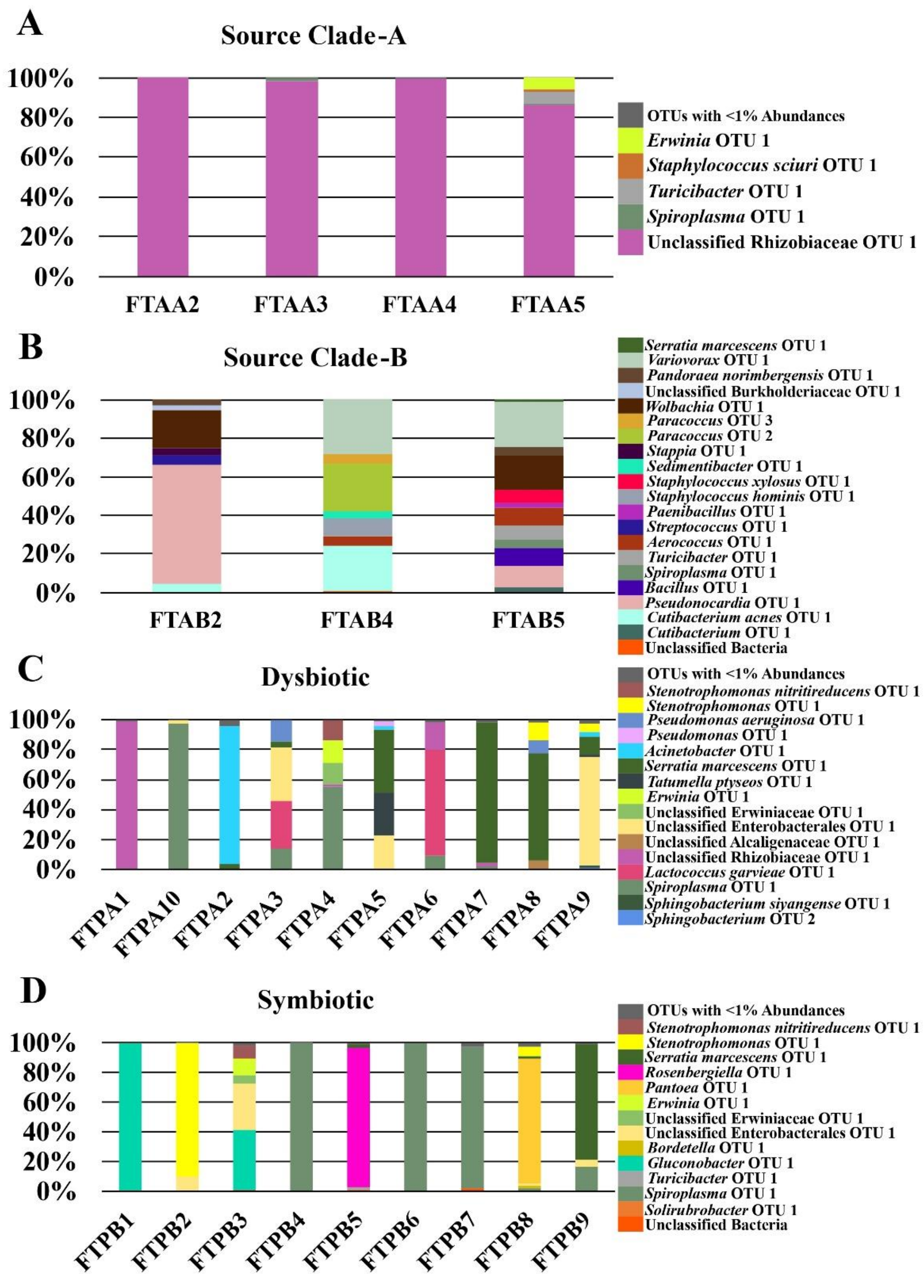

Figure S3. Taxonomic bar plots for each rarified bacteria microbiome sample of fungi associated with *Trachymyrmex pomonae* subcolonies and their fungal sources Taxonomic bar plots for each rarified sample of *Trachymyrmex pomonae* (A) source Clade-A fungi (n = 4), (B) source Clade-B fungi (n = 3), (C) dysbiotic, Clade-A fungi (n = 10), and (D) symbiotic, Clade-B fungi (n = 9). Bar plots are based on OTUs that occur in abundances greater than 1%. Sample identifiers correspond to those in Supplemental Table S1.

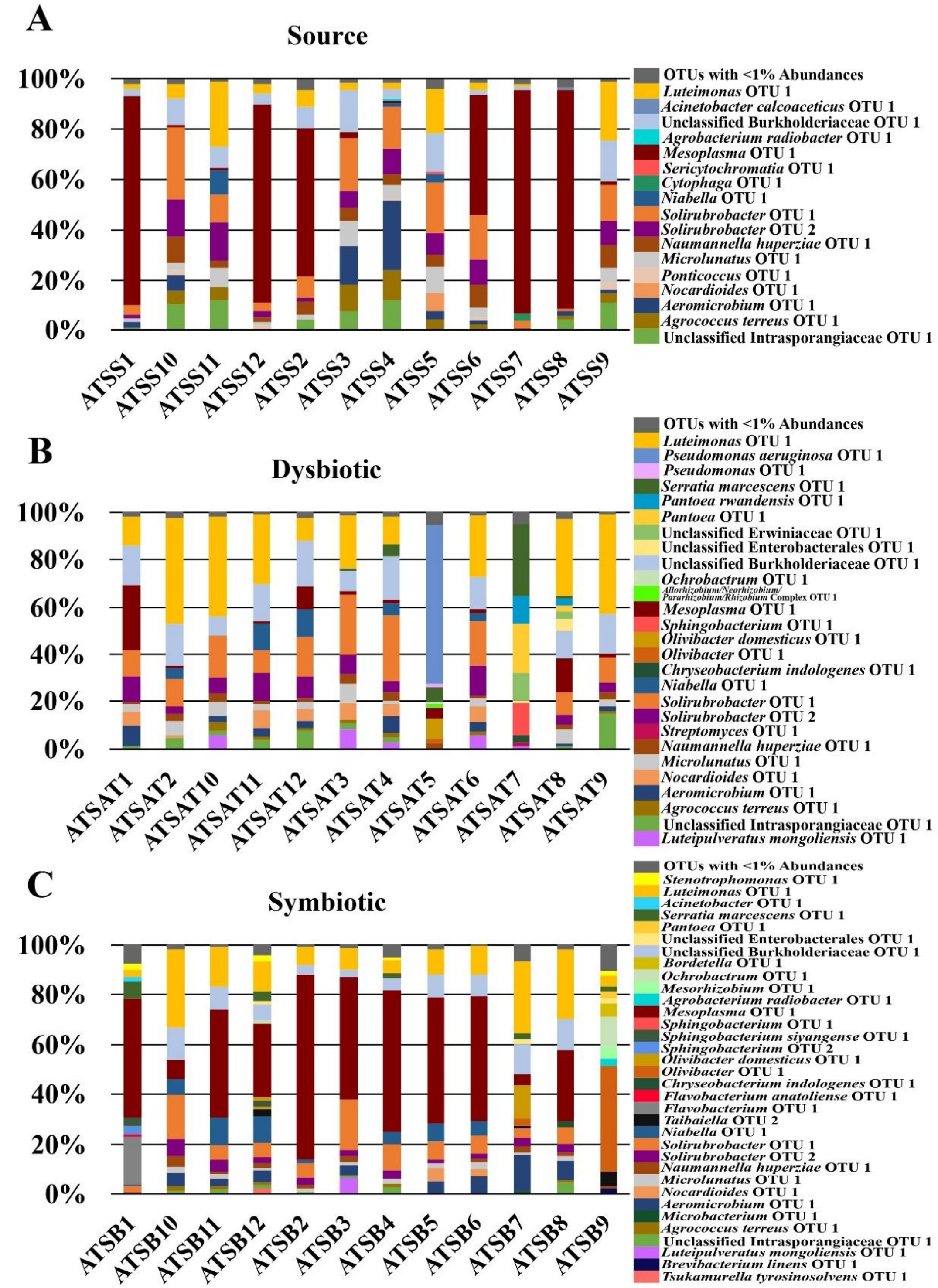

Figure S4. Taxonomic bar plots for each rarified bacteria microbiome sample of *Trachymyrmex septentrionalis* ants Taxonomic bar plots for each rarified sample of *Trachymyrmex septentrionalis* (A) source ants (n = 12), (B) dysbiotic, Clade-A growing ants (n = 12), and (C) symbiotic, Clade-B growing ants (n = 12). Bar plots are based on OTUs that occur in abundances greater than 1%. Sample identifiers correspond to those in Supplemental Table S1.

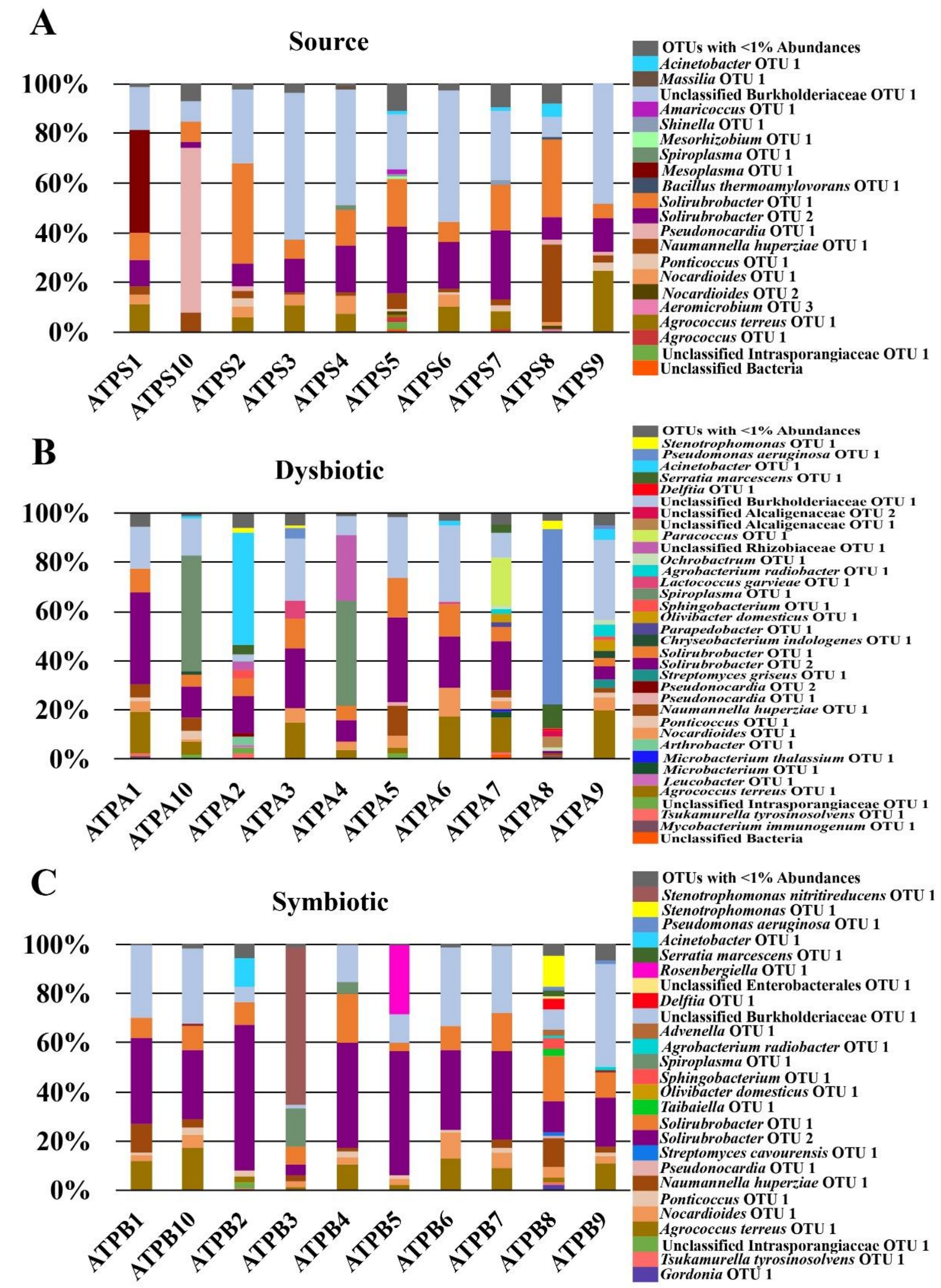

Figure S5. Taxonomic bar plots for each rarified bacteria microbiome sample of *Trachymyrmex pomonae* ants Taxonomic bar plots for each rarified sample of *Trachymyrmex pomonae* (A) source ants (n = 10), (B) dysbiotic, Clade-A growing ants (n = 10), and (C) symbiotic, Clade-B growing ants (n = 10). Bar plots are based on OTUs that occur in abundances greater than 1%. Sample identifiers correspond to those in Supplemental Table S1.

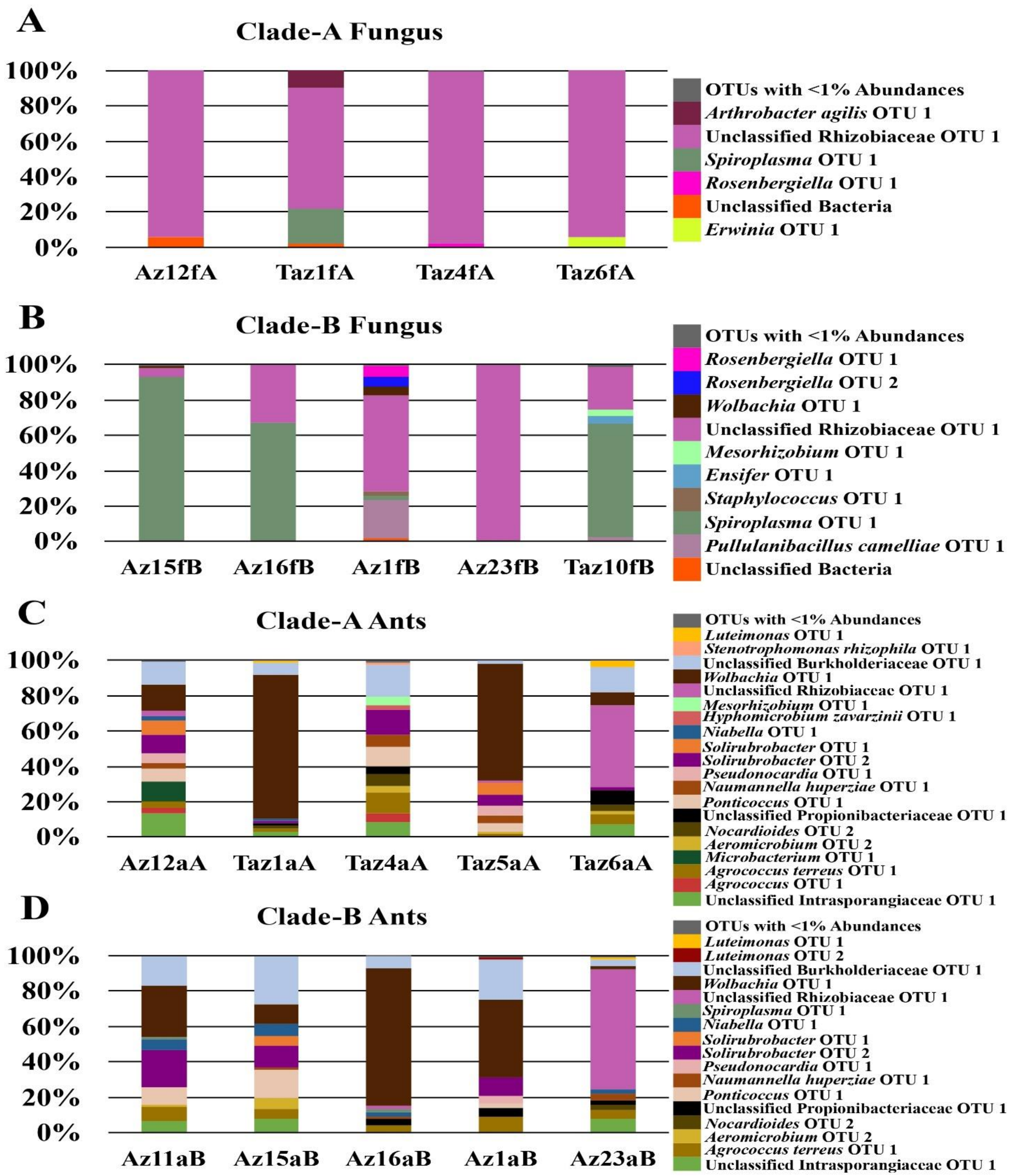

Figure S6. Taxonomic bar plots for each rarified microbiome sample of *Trachymyrmex arizonensis* fungi and ants Taxonomic bar plots for each rarified sample of *Trachymyrmex arizonensis* (A) Clade-A fungus (n = 4), (B) Clade-B fungus (n = 5), (C) Clade-A ants (n = 5), and (D) Clade-B ants (n = 5). Bar plots are based on OTUs that occur in abundances greater than 1%.

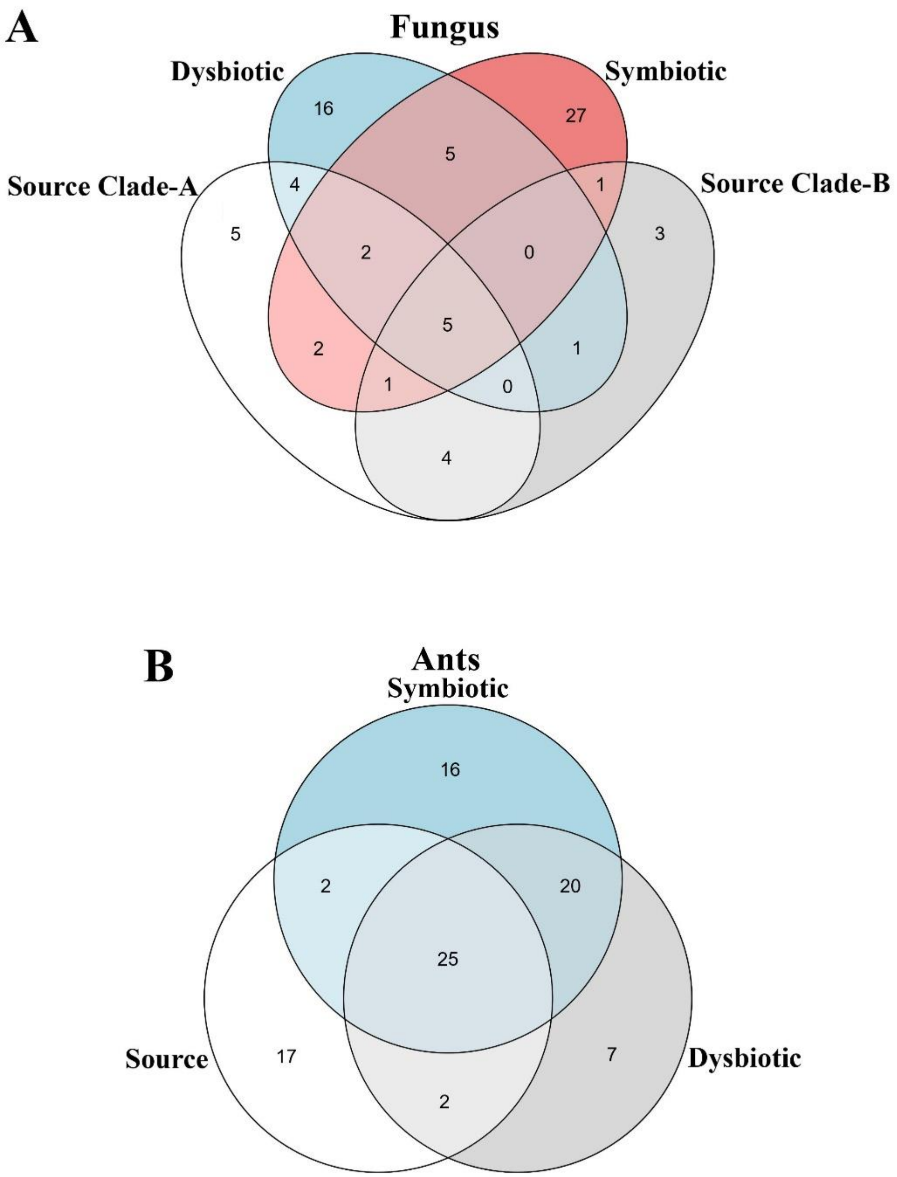

Figure S7. Venn diagrams of OTUs shared between *Trachymyrmex septentrionalis* associated ants and fungal gardens Venn diagrams illustrating shared OTUs between *T. septentrionalis* (A) associated fungi and (B) ant samples. Of the 76 bacterial OTUs found in the *T. septentrionalis* fungus gardens, five were found across all sample types. However, only *Mesoplasma* OTU 1 was found in abundances greater than 1% across each sample type. While dysbiotic gardens and symbiotic gardens additionally shared another five more OTUs, none were in abundances greater than 1%. Of the 89 OTUs found in all the *T. septentrionalis* ant microbiomes, 25 were found across each sample type. 11 of these OTUs were found in abundances greater than 1% and constitute the majority of the sequences found for each experimental group.

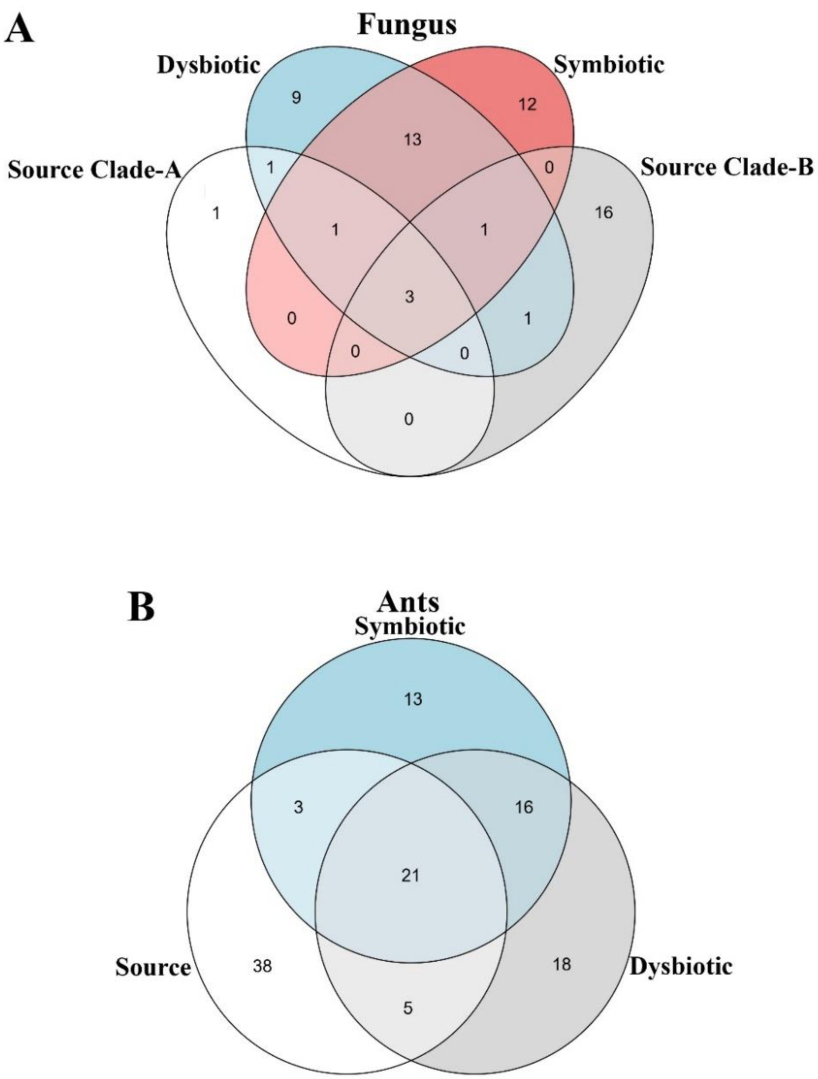

Figure S8. Venn diagrams of OTUs shared between *Trachymyrmex pomonae* associated ants and fungal gardens Venn diagrams illustrating shared OTUs between *T. pomonae* (A) associated fungi and (B) ant samples. Of the 58 OTUs found in the *T. pomonae* fungal microbiomes, only 3 were found within each sample type, but none of these OTUs were in percentages greater than 1% across all sample types. Notably, the dysbiotic Clade-A and symbiotic Clade-B samples uniquely shared thirteen OTUs, with *Spiroplasma* OTU 1, *Serratia marcescens* OTU 1, Unclassified Enterobacterales OTU 1, *Stenotrophomonas* OTU 1, and *Stenotrophomonas nitritireducens* OTU 1 being found in greater than 1% abundances in both sample types. Of the 114 OTUs found in the *T. pomonae* ant microbiomes, 21 were found within samples of each sample type. Of these 21 OTUs, 7 were found in abundances greater than 1% for each sample type. Dysbiotic ants growing Clade-A fungi and symbiotic ants growing Clade-B fungi also uniquely shared 16 OTUs, of which only 2 were found in abundances greater than 1% for each sample type.

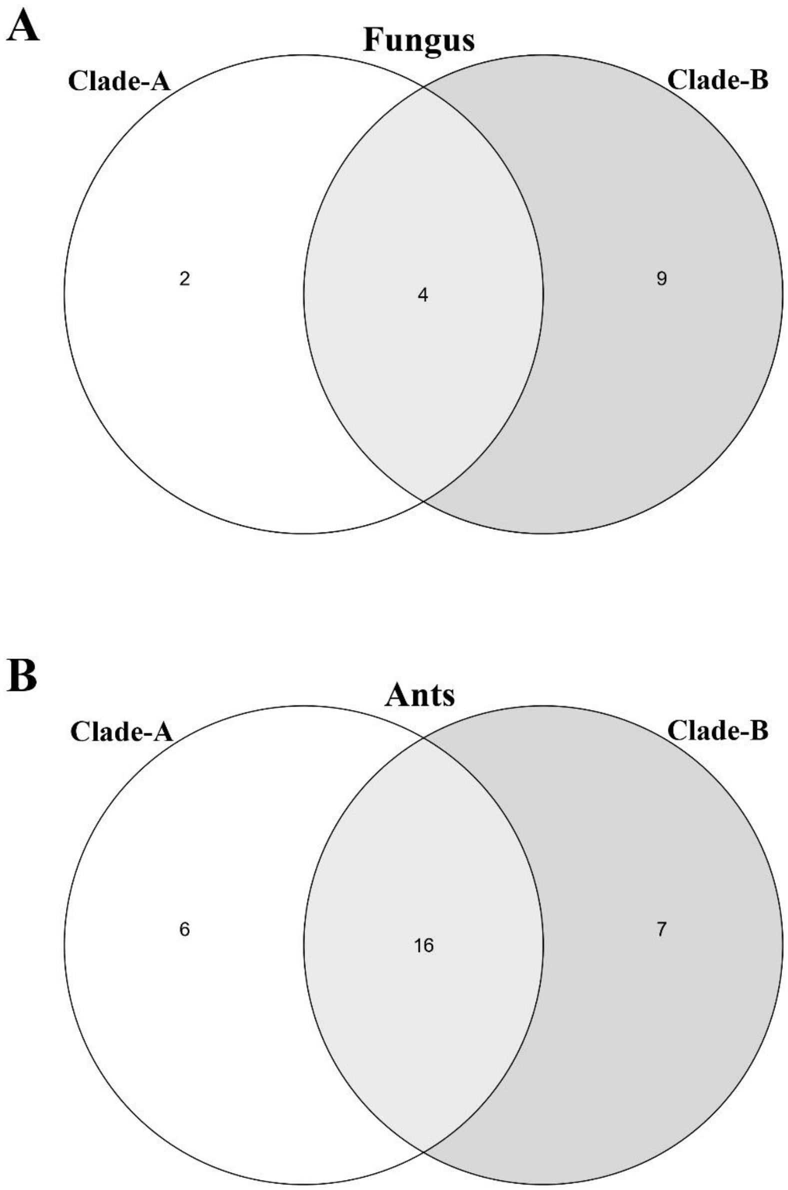

Figure S9. Venn diagrams of OTUs shared between *Trachymyrmex arizonensis* associated ants and fungal gardens Venn diagrams illustrating shared OTUs between *T. arizonensis* (A) associated fungi and (B) ant samples. Of the 29 OTUs found in the *T. arizonensis* ant microbiomes, 16 OTUs were found within both sample types, with only 11 found in abundances greater than 1% for each sample type. These 11 OTUs constitute the majority of the sequences found for both sample types. Of the 15 OTUs found in the *T. arizonensis* fungi microbiomes, 4 were found within both sample types. Only *Spiroplasma* OTU 1 and Unclassified Rhizobiaceae OTU 1 were found in abundances greater than 1% for each sample type, but these were the majority of the sequences found for both sample types.

Table S1. Number of workers utilized for each subcolony Initial number of workers placed in each *T. septentrionalis* (TS) or *T. pomonae* (TP) subcolony. Colony identifier corresponds to the original colony the subcolonies were derived from. Microbiome sample identifier corresponds to the individual microbiome samples (for ant and fungi) taken for downstream analysis. Collapse date corresponds to the date of the collapse of the fungal garden, where NA signifies the colony did not collapse.

Subcolony Species	Colony Identifier	Microbiome Sample Identifier (Ant / Fungus)	Initial Number of Workers	Collapse Date
TS	BB210408–1	ATSAT1 / FTSAT1	20	10/8/21
TS	BB210408–1	ATSB1 / FTSB1	20	8/11/21
TS	BB210408–2	ATSAT2 / FTSAT2	20	9/29/21
TS	BB210408–2	ATSB2 / FTSB2	20	NA
TS	BB210409–1	ATSAT3 / FTSAT3	20	9/29/21
TS	BB210409–1	ATSB3 / FTSB3	20	NA
TS	BB210409–2	ATSAT4 / FTSAT4	20	9/15/21
TS	BB210409–2	ATSB4 / FTSB4	20	8/11/21
TS	BB210409–3	ATSAT5 / FTSAT5	15	9/22/21
TS	BB210409–3	ATSB5 / FTSB5	15	10/6/21
TS	KB210408–1	ATSAT6 / FTSAT6	20	NA
TS	KB210408–1	ATSB6 / FTSB6	20	9/22/21
TS	KB210408–2	ATSAT7 / FTSAT7	20	9/1/21
TS	KB210408–2	ATSB7 / FTSB7	20	NA
TS	KB210408–3	ATSAT8 / FTSAT8	10	9/22/21
TS	KB210408–3	ATSB8 / FTSB8	10	NA
TS	KB210409–1	ATSAT9 / FTSAT9	20	10/6/21
TS	KB210409–1	ATSB9 / FTSB9	20	9/15/23
TS	KB210409–2	ATSAT10 / FTSAT10	20	10/6/21
TS	KB210409–2	ATSB10 / FTSB10	20	NA
TS	KB210409–3	ATSAT11 / FTSAT11	20	NA

TS	KB210409–3	ATSB11 / FTSB11	20	NA
TS	KB210409–4	ATSAT12 / FTSAT12	20	NA
TS	KB210409–4	ATSB12 / FTSB12	20	9/15/23
TP	CR190803–1B	ATPA1 / FTPA1	10	7/28/21
TP	CR190803–1B	ATPB1 / FTPB1	10	NA
TP	CVB190812–2	ATPA2 / FTPA2	20	7/21/21
TP	CVB190812–2	ATPB2 / FTPB2	20	NA
TP	JNS190801–1	ATPA3 / FTPA3	15	7/14/21
TP	JNS190801–1	ATPB3 / FTPB3	15	NA
TP	JNS190802–2	ATPA4 / FTPA4	20	7/28/21
TP	JNS190802–2	ATPB4 / FTPB4	20	NA
TP	JNS190802–3	ATPA5 / FTPA5	20	7/28/21
TP	JNS190802–3	ATPB5 / FTPB5	20	NA
TP	JNS190802–4	ATPA6 / FTPA6	15	7/21/23
TP	JNS190802–4	ATPB6 / FTPB6	15	NA
TP	JNS190802–6	ATPA7 / FTPA7	20	7/21/23
TP	JNS190802–6	ATPB7 / FTPB7	20	NA
TP	JNS190802–8	ATPA8 / FTPA8	5	7/14/21
TP	JNS190802–8	ATPB8 / FTPB8	5	NA
TP	JNS190802–9	ATPA9 / FTPA9	20	7/28/21
TP	JNS190802–9	ATPB9 / FTPB9	20	NA
TP	KK190802–1	ATPA10 / FTPA10	20	7/28/21
TP	KK190802–1	ATPB10 / FTPB10	20	NA

Table S2. Bray Curtis dissimilarity index between the fungal garden microbiomes of *Trachymyrmex septentrionalis* subcolonies derived from the same source colony Bray Curtis dissimilarity index between the fungal microbiomes of *Trachymyrmex septentrionalis* subcolonies from the same source colony, including subcolonies provided Clade-A fungus (dysbiotic) and subcolonies provided Clade-B fungus (symbiotic). Values closer to 1 signify greater dissimilarity between communities. Values with ***** refer to where the microbiome sample from either one of the gardens (dysbiotic or symbiotic) did not meet the sequence rarefaction threshold and was removed was the analysis.

**Colony: BB210408–1**		**Colony: BB210408–2**	
	**Symbiotic**		**Symbiotic**
**Dysbiotic**	0.03	**Dysbiotic**	0.75
**Colony: BB210409–1**		**Colony: BB210409–2**	
	**Symbiotic**		**Symbiotic**
**Dysbiotic**	*****	**Dysbiotic**	*****
**Colony: BB210409-3**		**Colony: KB210408–1**	
	**Symbiotic**		**Symbiotic**
**Dysbiotic**	0.71	**Dysbiotic**	0.02
**Colony: KB210408–2**		**Colony: KB210408–3**	
	**Symbiotic**		**Symbiotic**
**Dysbiotic**	0.90	**Dysbiotic**	*****
**Colony: KB210409–1**		**Colony: KB210409–2**	
	**Symbiotic**		**Symbiotic**
**Dysbiotic**	0.99	**Dysbiotic**	*****
**Colony: KB210409–3**		**Colony: KB210409–4**	
	**Symbiotic**		**Symbiotic**
**Dysbiotic**	*****	**Dysbiotic**	0.04

Table S3. Bray Curtis dissimilarity index between the fungal garden microbiomes of *Trachymyrmex pomonae* subcolonies derived from the same source colony Bray Curtis dissimilarity index between the fungal microbiomes of *Trachymyrmex pomonae* subcolonies from the same source colony, including subcolonies provided Clade-A fungus (dysbiotic) and subcolonies provided Clade-B fungus (symbiotic). Values closer to 1 signify greater dissimilarity between communities. Values with ***** refer to where the microbiome sample from either one of the gardens (dysbiotic or symbiotic) did not meet the sequence rarefaction threshold and was removed was the analysis.

**Colony: CR190803–1B**		**Colony: CVB190812–2**	
	**Symbiotic**		**Symbiotic**
**Dysbiotic**	1.0	**Dysbiotic**	0.99
**Colony: JNS190801–1**		**Colony: JNS190802–2**	
	**Symbiotic**		**Symbiotic**
**Dysbiotic**	0.66	**Dysbiotic**	0.44
**Colony: JNS190802–3**		**Colony: JNS190802–4**	
	**Symbiotic**		**Symbiotic**
**Dysbiotic**	0.97	**Dysbiotic**	0.91
**Colony: JNS190802–6**		**Colony: JNS190802–8**	
	**Symbiotic**		**Symbiotic**
**Dysbiotic**	0.98	**Dysbiotic**	0.91
**Colony: JNS190802–9**		**Colony: KK190802–1**	
	**Symbiotic**		**Symbiotic**
**Dysbiotic**	0.82	**Dysbiotic**	*****

Table S4. Bray Curtis dissimilarity index between the microbiomes of *Trachymyrmex septentrionalis* ants derived from the same source colony Bray Curtis dissimilarity index between the microbiomes of *Trachymyrmex septentrionalis* ants from the same source colony, including the source ants as well as ants provided Clade-A fungus (dysbiotic) and ants provided Clade-B fungus (symbiotic). Values closer to 1 signify greater dissimilarity between communities.

**Colony: BB210408–1**			**Colony: BB210408–2**		
	**Source**	**Dysbiotic**		**Source**	**Dysbiotic**
**Dysbiotic**	0.56		**Dysbiotic**	0.54	
**Symbiotic**	0.45	0.64	**Symbiotic**	0.19	0.65
**Colony: BB210409–1**			**Colony: BB210409–2**		
	**Source**	**Dysbiotic**		**Source**	**Dysbiotic**
**Dysbiotic**	0.41		**Dysbiotic**	0.53	
**Symbiotic**	0.58	0.51	**Symbiotic**	0.70	0.60
**Colony: BB210409-3**			**Colony: KB210408–1**		
	**Source**	**Dysbiotic**		**Source**	**Dysbiotic**
**Dysbiotic**	0.96		**Dysbiotic**	0.58	
**Symbiotic**	0.55	0.95	**Symbiotic**	0.32	0.54
**Colony: KB210408–2**			**Colony: KB210408–3**		
	**Source**	**Dysbiotic**		**Source**	**Dysbiotic**
**Dysbiotic**	0.99		**Dysbiotic**	0.78	
**Symbiotic**	0.88	0.92	**Symbiotic**	0.60	0.28
**Colony: KB210409–1**			**Colony: KB210409–2**		
	**Source**	**Dysbiotic**		**Source**	**Dysbiotic**
**Dysbiotic**	0.23		**Dysbiotic**	0.56	
**Symbiotic**	0.90	0.90	**Symbiotic**	0.42	0.27
**Colony: KB210409–3**			**Colony: KB210409–4**		
	**Source**	**Dysbiotic**		**Source**	**Dysbiotic**
**Dysbiotic**	0.32		**Dysbiotic**	0.80	
**Symbiotic**	0.48	0.56	**Symbiotic**	0.51	0.53

Table S5. Bray Curtis dissimilarity index between the microbiomes of *Trachymyrmex pomonae* ants derived from the same source colony Bray Curtis dissimilarity index between the microbiomes of *Trachymyrmex pomonae* ants from the same source colony, including the source ants as well as ants provided Clade-A fungus (dysbiotic) and ants provided Clade-B fungus (symbiotic). Values closer to 1 signify greater dissimilarity between communities.

**Colony: CR190803–1B**			**Colony: CVB190812–2**		
	**Source**	**Dysbiotic**		**Source**	**Dysbiotic**
**Dysbiotic**	0.44		**Dysbiotic**	0.79	
**Symbiotic**	0.46	0.19	**Symbiotic**	0.69	0.58
**Colony: JNS190801–1**			**Colony: JNS190802–2**		
	**Source**	**Dysbiotic**		**Source**	**Dysbiotic**
**Dysbiotic**	0.37		**Dysbiotic**	0.68	
**Symbiotic**	0.81	0.82	**Symbiotic**	0.38	0.65
**Colony: JNS190802–3**			**Colony: JNS190802–4**		
	**Source**	**Dysbiotic**		**Source**	**Dysbiotic**
**Dysbiotic**	0.24		**Dysbiotic**	0.26	
**Symbiotic**	0.55	0.45	**Symbiotic**	0.24	0.14
**Colony: JNS190802–6**			**Colony: JNS190802–8**		
	**Source**	**Dysbiotic**		**Source**	**Dysbiotic**
**Dysbiotic**	0.50		**Dysbiotic**	0.96	
**Symbiotic**	0.17	0.47	**Symbiotic**	0.46	0.87
**Colony: JNS190802–9**			**Colony: KK190802–1**		
	**Source**	**Dysbiotic**		**Source**	**Dysbiotic**
**Dysbiotic**	0.35		**Dysbiotic**	0.77	
**Symbiotic**	0.24	0.37	**Symbiotic**	0.76	0.54

Table S6. Mean percentage of each major OTU comprising each fungus sample type OTUs with mean sample type abundance percentages greater than 1%, along with their corresponding mean percentage and standard deviation (SD), for source Clade-A, dysbiotic (grown Clade-A gardens), source Clade-B, and symbiotic (grown Clade-B gardens) fungus for *T. septentrionalis* and *T. pomonae*. Additionally, mean sample type abundance percentages of *T. arizonensis* Clade-A and Clade-B fungi are presented.

T. septentrionalis							
**Source Clade-A**		**Dysbiotic**		**Source Clade-B**		**Symbiotic**	
**OTU**	**Mean Percentage ± SD**	**OTU**	**Mean Percentage ± SD**	**OTU**	**Mean Percentage ± SD**	**OTU**	**Mean Percentage ± SD**
Pantoea OTU 1	65.29 ± 20.05	*Mesoplasma* OTU 1	51.39 ± 41.87	*Mesoplasma* OTU 1	99.00 ± 0.56	*Mesoplasma* OTU 1	95.38 ± 6.87
Pseudomonas OTU 1	8.86 ± 12.53	*Serratia marcescens* OTU 1	15.88 ± 33.54				
Mesoplasma OTU 1	8.19 ± 6.51	Unclassified Enterobacterales OTU 1	10.76 ± 22.13				
Erwinia OTU 2	5.95 ± 8.42	*Pantoea rwandensis* OTU 1	5.84 ± 14.25				
Raoultella OTU 1	3.67 ± 5.19	*Pantoea* OTU 1	4.02 ± 9.25				
Ewingella americana OTU 1	1.48 ± 2.09	*Sphingobacterium* OTU 1	3.65 ± 8.60				
Unclassified Burkholderiaceae OTU 1	1.14 ± 1.14	Unclassified Erwiniaceae OTU 1	2.88 ± 4.52				
Luteimonas OTU 1	1.05 ± 0.58	Unclassified Enterobacteriaceae OTU 1	1.41 ± 3.45				
		Erwinia OTU 2	1.06 ± 2.60				
T. pomonae							
**Source Clade-A**		**Dysbiotic**		**Source Clade-B**		**Symbiotic**	
**OTU**	**Mean Percentage ± SD**	**OTU**	**Mean Percentage ± SD**	**OTU**	**Mean Percentage ± SD**	**OTU**	**Mean Percentage ± SD**
Unclassified Rhizobiaceae OTU 1	95.86 ± 5.70	*Serratia marcescens* OTU 1	22.71 ± 32.76	*Pseudonocardia* OTU 1	24.21 ± 26.81	*Spiroplasma* OTU 1	35.06 ± 45.00
Erwinia OTU 1	1.51 ± 2.62	*Spiroplasma* OTU 1	17.74 ± 31.22	*Variovorax* OTU 1	17.28 ± 12.39	*Gluconobacter* OTU 1	15.47 ± 32.29
Turicibacter OTU 1	1.38 ± 2.39	Unclassified Enterobacterales OTU 1	13.42 ± 23.24	*Wolbachia* OTU 1	12.54 ± 8.90	*Stenotrophomonas* OTU 1	10.82 ± 27.89

		Unclassified Rhizobiaceae OTU 1	12.34 ± 29.61	*Cutibacterium acnes* OTU 1	9.21 ± 10.03	*Rosenbergiella* OTU 1	10.41 ± 29.44
		Lactococcus garvieae OTU 1	10.26 ± 22.27	*Paracoccus* OTU 2	8.16 ± 11.54	*Pantoea* OTU 1	9.24 ± 26.13
		Acinetobacter OTU 1	9.89 ± 27.71	*Aerococcus* OTU 1	4.65 ± 3.66	*Serratia marcescens* OTU 1	9.06 ± 24.17
		Tatumella ptyseos OTU 1	3.03 ± 8.65	*Bacillus* OTU 1	3.07 ± 4.34	Unclassified Enterobacterales OTU 1	5.44 ± 9.91
		Pseudomonas aeruginosa OTU 1	2.32 ± 4.81	*Staphylococcus hominis* OTU 1	2.98 ± 4.22	*Erwinia* OTU 1	1.17 ± 3.31
		Stenotrophomonas OTU 1	2.00 ± 3.99	*Turicibacter* OTU 1	2.54 ± 3.60	*Stenotrophomonas nitritireducens* OTU 1	1.05 ± 2.81
		Erwinia OTU 1	1.50 ± 4.50	*Pandoraea norimbergensis* OTU 1	2.37 ± 1.84		
		Unclassified Erwiniaceae OTU 1	1.47 ± 4.42	*Staphylococcus xylosus* OTU 1	2.28 ± 3.23		
		Stenotrophomonas nitritireducens OTU 1	1.39 ± 4.11	*Streptococcus* OTU 1	1.67 ± 2.36		
				Paracoccus OTU 1	1.58 ± 2.23		
				Spiroplasma OTU 1	1.49 ± 2.11		
				Sedimentibacter OTU 1	1.40 ± 1.98		
				Stappia OTU 1	1.23 ± 1.74		

T. arizonensis			
**Clade-A**		**Clade-B**	
**OTU**	**Mean Percentage ± SD**	**OTU**	**Mean Percentage ± SD**
Unclassified Rhizobiaceae OTU 1	88.60 ± 11.95	*Spiroplasma* OTU 1	45.38 ± 37.25
Spiroplasma OTU 1	4.95 ± 8.57	Unclassified Rhizobiaceae OTU 1	43.24 ± 32.18
Arthrobacter agilis OTU 1	2.5 ± 4.33	*Pullulanibacillus camelliae* OTU 1	4.92 ± 8.64
Unclassified Bacteria	2.05 ± 2.25	*Wolbachia* OTU 1	1.22 ± 1.90
Erwinia OTU 1	1.38 ± 2.38	*Rosenbergiella* OTU 1	1.20 ±2.40
		Rosenbergiella OTU 2	1.12 ± 2.24

Table S7. Indicator species analysis on fungal gardens for each ant species The OTUs with significant (p-value < 0.05) indicator values (IVs) when comparing the microbiomes of source Clade-A, dysbiosis (grown Clade-A fungi), source Clade-B, and symbiosis (grown Clade-B fungi) fungus samples using an indicator species analysis (ISA) for *T. septentrionalis* and *T. pomonae*. Additionally, results comparing *T. arizonensis* Clade-A and Clade-B fungi are presented. Each analysis utilized 9,999 permutations. OTUs found to be the indicator of a combination of multiple sample types are indicated with superscripts ([a]) and are listed with each sample type. While *T. arizonensis* had no indicator taxa, *T. pomonae* and *T. septentrionalis* had indicator taxa; with notable taxa being Unclassified Rhizobiaceae OTU 1 being associated with source Clade-A *T. pomonae*, *Pantoea* OTU 1 associated with source Clade-A *T. septentrionalis*, and *Mesoplasma* OTU 1 associated with both source Clade-B and symbiosis *T. septentrionalis*.

T. septentrionalis							
**Source Clade-A**		**Dysbiosis**		**Source Clade-B**		**Symbiosis**	
**OTU**	**IV, p-value**	**OTU**	**IV, p-value**	**OTU**	**IV, p-value**	**OTU**	**IV, p-value**
Pantoea OTU 1	0.928, 0.0004	No indicator species	-	*Mesoplasma* OTU 1[a]	0.788, 0.0027	*Mesoplasma* OTU 1[a]	0.788, 0.0027
Luteimonas OTU 1	0.903, 0.0006						
Solirubrobacter OTU 1	0.884, 0.0004						
Naumannella huperziae OTU 1	0.813, 0.0010						
Aeromicrobium OTU 1	0.731, 0.0006						
Unclassified Burkholderiaceae OTU 1	0.701, 0.0006						
Niabella OTU 1	0.620, 0.0174						
Pseudomonas OTU 1	0.520, 0.0373						
T. pomonae							
**Source Clade-A**		**Dysbiosis**		**Source Clade-B**		**Symbiosis**	
**OTU**	**IV, p-value**	**OTU**	**IV, p-value**	**OTU**	**IV, p-value**	**OTU**	**IV, p-value**
Unclassified Rhizobiaceae OTU 1	0.929, 0.0004	No indicator species	-	*Wolbachia* OTU 1	0.773, 0.0092	No indicator species	-
				Variovorax OTU 1	0.770, 0.0077		

				Pandoraea norimbergensis OTU 1	0.745, 0.0092		
				Aerococcus OTU 1	0.740, 0.0077		
				Cutibacterium acnes OTU 1	0.622, 0.0103		
				Pseudonocardia OTU 1	0.616, 0.0044		
T. arizonensis							
**Clade-A**				**Clade-B**			
**OTU**		**IV, p-value**		**OTU**		**IV, p-value**	
No indicator species		-		No indicator species		-	

Table S8. PERMANOVA results for ant comparisons Results of PERMANOVA tests between the different sample types for *T. septentrionalis* and *T. pomonae* ants. Every PERMANOVA test utilized Bray Curtis distances and had 9,999 permutations. Bolded statistics mean the bacterial microbiomes of the sample types were found to be significantly different, or p < 0.05.

T. septentrionalis			
	**Source**	**Dysbiotic**	**Symbiotic**
**Source**			
**Dysbiotic**	**$F = 3.89$, $R^2 = 0.15$, $p = 0.012$**		
**Symbiotic**	$F = 2.19$, R2 = 0.09, $p = 0.087$	**$F = 4.77$, $R^2 = 0.18$, $p = 0.001$**	
T. pomonae			
	**Source**	**Dysbiotic**	**Symbiotic**
**Source**			
**Dysbiotic**	**$F = 1.83$, $R^2 = 0.09$, $p = 0.024$**		
**Symbiotic**	**$F = 2.04$, $R^2 = 0.10$, $p = 0.040$**	$F = 1.22$, $R^2 = 0.06$, $p = 0.235$	

Table S9. Mean percentage of each major OTU comprising each ant sample type OTUs with mean sample type abundance percentages greater than 1%, along with their corresponding mean percentage and standard deviation (SD), for source, dysbiotic (ants provided Clade-A fungus), and symbiotic (ants provided Clade-B fungus) ants for *T. septentrionalis* and *T. pomonae*. Additionally, mean sample type abundance percentages of *T. arizonensis* ants growing Clade-A and Clade-B fungi are presented.

T. septentrionalis					
**Source**		**Dysbiotic**		**Symbiotic**	
**OTU**	**Mean Percentage ± SD**	**OTU**	**Mean Percentage ± SD**	**OTU**	**Mean Percentage ± SD**
Mesoplasma OTU 1	37.69 ± 38.03	*Luteimonas* OTU 1	22.70 ± 15.33	*Mesoplasma* OTU 1	36.83 ± 22.13
Solirubrobacter OTU 1	12.43 ± 8.32	*Solirubrobacter* OTU 1	13.24 ± 8.18	*Luteimonas* OTU 1	13.83 ± 9.60
Luteimonas OTU 1	7.87 ± 8.63	Unclassified Burkholderiaceae OTU 1	12.30 ± 6.48	*Solirubrobacter* OTU 1	7.88 ± 5.70
Unclassified Burkholderiaceae OTU 1	7.51 ± 5.73	*Solirubrobacter* OTU 2	6.24 ± 4.10	Unclassified Burkholderiaceae OTU 1	6.92 ± 4.42
Solirubrobacter OTU 2	6.83 ± 5.35	*Pseudomonas aeruginosa* OTU 1	5.67 ± 18.60	*Aeromicrobium* OTU 1	4.35 ± 4.11
Unclassified Intrasporangiaceae OTU 1	5.38 ± 4.92	*Mesoplasma* OTU 1	5.38 ± 7.93	*Niabella* OTU 1	4.00 ± 4.05
Aeromicrobium OTU 1	5.04 ± 7.94	*Microlunatus* OTU 1	3.74 ± 2.44	*Olivibacter* OTU 1	3.81 ± 11.76
Naumannella huperziae OTU 1	4.63 ± 3.38	*Serratia marcescens* OTU 1	3.63 ± 8.32	*Solirubrobacter* OTU 2	2.71 ± 1.78
Microlunatus OTU 1	4.29 ± 3.46	Unclassified Intrasporangiaceae OTU 1	3.39 ± 4.10	*Flavobacterium* OTU 1	1.62 ± 5.37
Agrococcus terreus OTU 1	3.83 ± 3.84	*Nocardioides* OTU 1	3.18 ± 3.10	*Naumannella huperziae* OTU 1	1.61 ± 1.11
Niabella OTU 1	1.24 ± 2.68	*Niabella* OTU 1	3.08 ± 4.22	*Microlunatus* OTU 1	1.60 ± 0.84
		Aeromicrobium OTU 1	2.66 ± 2.76	*Serratia marcescens* OTU 1	1.44 ± 2.07
		Naumannella huperziae OTU 1	2.11 ± 1.10	Unclassified Intrasporangiaceae OTU 1	1.31 ± 3.74
		Luteipulveratus mongoliensis OTU 1	2.08 ± 2.87	*Olivibacter domesticus* OTU 1	1.24 ± 1.35
		Pantoea OTU 1	1.96 ± 5.63	*Ochrobactrum* OTU 1	1.17 ± 3.34
		Sphingobacterium OTU 1	1.24 ± 3.27		

		Unclassified Erwiniaceae OTU 1	1.23 ± 3.26		
		Pantoea rwandensis OTU 1	1.21 ± 3.83		
		Agrococcus terreus OTU 1	1.15 ± 0.99		
T. pomonae					
**Source**		**Dysbiotic**		**Symbiotic**	
**OTU**	**Mean Percentage ± SD**	**OTU**	**Mean Percentage ± SD**	**OTU**	**Mean Percentage ± SD**
Unclassified Burkholderiaceae OTU 1	31.97 ± 17.59	*Solirubrobacter* OTU 2	18.11 ± 11.28	*Solirubrobacter* OTU 2	32.03 ± 15.71
Solirubrobacter OTU 1	16.37 ± 10.81	Unclassified Burkholderiaceae OTU 1	16.61 ± 10.84	Unclassified Burkholderiaceae OTU 1	20.37 ± 12.64
Solirubrobacter OTU 2	15.05 ± 7.58	*Agrococcus terreus* OTU 1	9.45 ± 7.33	*Solirubrobacter* OTU 1	11.08 ± 5.00
Agrococcus terreus OTU 1	8.08 ± 6.97	*Spiroplasma* OTU 1	8.97 ± 17.92	*Agrococcus terreus* OTU 1	8.05 ± 5.39
Pseudonocardia OTU 1	7.32 ± 19.74	*Solirubrobacter* OTU 1	7.82 ± 4.64	*Stenotrophomonas nitritireducens* OTU 1	6.37 ± 19.11
Naumannella huperziae OTU 1	6.11 ± 8.66	*Pseudomonas aeruginosa* OTU 1	7.71 ± 21.25	*Nocardioides* OTU 1	4.00 ± 2.68
Mesoplasma OTU 1	4.13 ± 12.39	*Acinetobacter* OTU 1	5.42 ± 13.47	*Naumannella huperziae* OTU 1	3.74 ± 4.22
Nocardioides OTU 1	2.66 ± 2.49	*Nocardioides* OTU 1	4.11 ± 3.31	*Rosenbergiella* OTU 1	2.79 ± 8.37
Ponticoccus OTU 1	1.16 ± 1.38	Unclassified Rhizobiaceae OTU 1	3.11 ± 8.01	*Spiroplasma* OTU 1	2.05 ± 4.72
		Naumannella huperziae OTU 1	3.00 ± 3.68	*Ponticoccus* OTU 1	1.50 ± 0.88
		Paracoccus OTU 1	2.03 ± 5.76	*Stenotrophomonas* OTU 1	1.37 ± 3.71
		Serratia marcescens OTU 1	1.76 ± 2.96	*Acinetobacter* OTU 1	1.26 ± 3.39
		Ponticoccus OTU 1	1.00 ± 1.07		

T. arizonensis			
**Clade-A**		**Clade-B**	
**OTU**	**Mean Percentage ± SD**	**OTU**	**Mean Percentage ± SD**
Wolbachia OTU 1	33.96 ± 33.18	*Wolbachia* OTU 1	32.78 ± 26.70

Unclassified Burkholderiaceae OTU 1	10.94 ± 5.78	Unclassified Burkholderiaceae OTU 1	15.50 ± 9.13
Unclassified Rhizobiaceae OTU 1	10.12 ± 18.08	Unclassified Rhizobiaceae OTU 1	14.00 ± 27.11
Solirubrobacter OTU 2	6.66 ± 5.03	*Solirubrobacter* OTU 2	8.90 ± 8.10
Unclassified Intrasporangiaceae OTU 1	6.54 ± 4.70	*Agrococcus terreus* OTU 1	6.40 ± 1.76
Agrococcus terreus OTU 1	4.92 ± 3.67	*Ponticoccus* OTU 1	5.46 ± 6.20
Ponticoccus OTU 1	4.78 ± 4.35	Unclassified Intrasporangiaceae OTU 1	4.54 ± 3.73
Solirubrobacter OTU 1	3.02 ± 3.71	*Niabella* OTU 1	3.34 ± 2.38
Naumannella huperziae OTU 1	2.80 ± 2.64	Unclassified Propionibacteriaceae OTU 1	2.26 ± 2.04
Unclassified Propionibacteriaceae OTU 1	2.70 ± 3.06	*Aeromicrobium* OTU 2	1.60 ± 2.40
Nocardioides OTU 2	2.40 ± 2.47	*Naumannella huperziae* OTU 1	1.20 ± 1.28
Pseudonocardia OTU 1	2.24 ± 2.75	*Solirubrobacter* OTU 1	1.14 ± 2.28
Microbacterium OTU 1	2.16 ± 4.32		
Agrococcus OTU 1	1.60 ± 2.15		
Aeromicrobium OTU 2	1.42 ± 1.35		
Mesorhizobium OTU 1	1.00 ± 2.00		
Luteimonas OTU 1	1.00 ± 1.38		

Table S10. Indicator species analysis for each ant species The OTUs with significant (p-value < 0.05) indicator values (IVs) when comparing the microbiomes of source, dysbiosis (Clade-A provided ants), and symbiosis (Clade-B provided ants) ant samples using an indicator species analysis (ISA) for *T. septentrionalis* and *T. pomonae*. Additionally, results comparing *T. arizonensis* ants growing Clade-A and Clade-B fungi are presented. Each analysis utilized 9,999 permutations. OTUs found to be the indicator of a combination of two out the three sample types are indicated with superscripts ([a],[b],[c]) and are listed with each sample type. While *T. arizonensis* had no indicator taxa, *T. pomonae* and *T. septentrionalis* had indicator taxa for each sample type; with notable taxa being *Pseudonocardia* OTU 1 being associated with source *T. pomonae*, *Serratia marcescens* OTU 1 associated with dysbiosis *T. pomonae*, and *Mesoplasma* OTU 1 associated with both source and symbiosis *T. septentrionalis*.

T. septentrionalis					
**Source**		**Dysbiosis**		**Symbiosis**	
**OTU**	**IV, p-value**	**OTU**	**IV, p-value**	**OTU**	**IV, p-value**
Naumannella huperziae OTU 1	0.530, 0.0034	*Nocardioides* OTU 1	0.443, 0.0193	*Mesoplasma* OTU 1[a]	0.504, 0.0057
Agrococcus terreus OTU 1	0.518, 0.0018	*Luteimonas* OTU 1	0.428, 0.0240	*Stenotrophomonas* OTU 1	0.471, 0.0158
Ponticoccus OTU 1	0.515, 0.0028	*Microlunatus* OTU 1[b]	0.422, 0.0307	*Sphingobacterium siyangense* OTU 1	0.363, 0.0259
Mesoplasma OTU 1[a]	0.504, 0.0057	*Solirubrobacter* OTU 2[c]	0.413, 0.0361		
Microlunatus OTU 1[b]	0.422, 0.0307	Unclassified Burkholderiaceae OTU 1	0.397, 0.0467		
Solirubrobacter OTU 2[c]	0.413, 0.0361	*Pseudomonas aeruginosa* OTU 1	0.241, 0.0215		
T. pomonae					
**Source**		**Dysbiosis**		**Symbiosis**	
**OTU**	**IV, p-value**	**OTU**	**IV, p-value**	**OTU**	**IV, p-value**
Flavobacterium OTU 1	0.516, 0.0222	*Pseudonocardia* OTU 2	0.522, 0.0232	*Solirubrobacter* OTU 2	0.517, 0.0091
Shinella OTU 1	0.496, 0.0231	*Ochrobactrum* OTU 1	0.514, 0.0097		
Pseudonocardia OTU 1	0.282, 0.0134	*Serratia marcescens* OTU 1	0.405, 0.0376		
		Chryseobacterium indologenes OTU 1	0.405, 0.0223		

T. arizonensis			
**Clade-A**		**Clade-B**	
**OTU**	**IV, p-value**	**OTU**	**IV, p-value**
No indicator species	-	No indicator species	-

Supplemental References

1. Seal, J. N., Schiøtt, M. & Mueller, U. G. Ant-fungus species combinations engineer physiological activity of fungus gardens. *J. Exp. Biol.* **217**, 2540–2547 (2014).

2. Rabeling, C., Cover, S. P., Johnson, R. A. & Mueller, U. G. A review of the North American species of the fungus-gardening ant genus *Trachymyrmex* (Hymenoptera: Formicidae). *Zootaxa* 1–53 (2007). doi:10.5281/zenodo.180014

3. Mueller, U. G. *et al.* Phylogenetic patterns of ant–fungus associations indicate that farming strategies, not only a superior fungal cultivar, explain the ecological success of leafcutter ants. *Mol. Ecol.* **27**, 2414–2434 (2018).

4. Wheeler, W. M. The fungus–growing ants of North America. *Bull. Am. Museum Nat. Hist.* **23**, 669–807 (1907).

5. Bollazzi, M. & Roces, F. Thermal preference for fungus culturing and brood location by workers of the thatching grass-cutting ant *Acromyrmex heyeri*. *Insectes Soc.* **49**, 153–157 (2002).

6. Kooij, P. W., Pullens, J. W. M., Boomsma, J. J. & Schiøtt, M. Ant mediated redistribution of a xyloglucanase enzyme in fungus gardens of *Acromyrmex echinatior*. *BMC Microbiol.* **16**, 81 (2016).

7. Rodrigues, A., Mueller, U. G., Ishak, H. D., Bacci, M. & Pagnocca, F. C. Ecology of microfungal communities in gardens of fungus-growing ants (Hymenoptera: Formicidae): A year-long survey of three species of attine ants in Central Texas. *FEMS Microbiol. Ecol.* **78**, 244–255 (2011).

8. Seal, J. N., Gus, J. & Mueller, U. G. Fungus-gardening ants prefer native fungal species: Do ants control their crops? *Behav. Ecol.* **23**, 1250–1256 (2012).

9. Kellner, K. *et al.* Co-evolutionary patterns and diversification of ant-fungus associations in the asexual fungus-farming ant *Mycocepurus smithii* in Panama. *J. Evol. Biol.* **26**, 1353–1362 (2013).

10. Mueller, U. G., Rehner, S. A. & Schultz, T. R. The Evolution of Agriculture in Ants. *Science* **281**, 2034–2038 (1998).

11. Kearse, M. *et al.* Geneious Basic: An integrated and extendable desktop software platform for the organization and analysis of sequence data. *Bioinformatics* **28**, 1647–1649 (2012).

12. Luiso, J., Kellner, K., Matthews, A. E., Mueller, U. G. & Seal, J. N. High diversity and multiple invasions to North America by fungi grown by the northern-most *Trachymyrmex* and *Mycetomoellerius* ant species. *Fungal Ecol.* **44**, 100878 (2020).

13. Rowan, C. D. Comparative microbiome analysis of the fungus gardening ant species *Trachymyrmex arizonensis*. (University of Texas at Tyler, 2021).
